# Non-linear Memristive Synaptic Dynamics for Efficient Unsupervised Learning in Spiking Neural Networks

**DOI:** 10.3389/fnins.2021.580909

**Published:** 2021-02-01

**Authors:** Stefano Brivio, Denys R. B. Ly, Elisa Vianello, Sabina Spiga

**Affiliations:** ^1^CNR - IMM, Unit of Agrate Brianza, Agrate Brianza, Italy; ^2^Université Grenoble Alpes, CEA, Leti, Grenoble, France

**Keywords:** spiking neural network, MNIST, neuromorphic, analog memory, STDP, memristive synapse, memristor, memristive devices

## Abstract

Spiking neural networks (SNNs) are a computational tool in which the information is coded into spikes, as in some parts of the brain, differently from conventional neural networks (NNs) that compute over real-numbers. Therefore, SNNs can implement intelligent information extraction in real-time at the edge of data acquisition and correspond to a complementary solution to conventional NNs working for cloud-computing. Both NN classes face hardware constraints due to limited computing parallelism and separation of logic and memory. Emerging memory devices, like resistive switching memories, phase change memories, or memristive devices in general are strong candidates to remove these hurdles for NN applications. The well-established training procedures of conventional NNs helped in defining the desiderata for memristive device dynamics implementing synaptic units. The generally agreed requirements are a linear evolution of memristive conductance upon stimulation with train of identical pulses and a symmetric conductance change for conductance increase and decrease. Conversely, little work has been done to understand the main properties of memristive devices supporting efficient SNN operation. The reason lies in the lack of a background theory for their training. As a consequence, requirements for NNs have been taken as a reference to develop memristive devices for SNNs. In the present work, we show that, for efficient CMOS/memristive SNNs, the requirements for synaptic memristive dynamics are very different from the needs of a conventional NN. System-level simulations of a SNN trained to classify hand-written digit images through a spike timing dependent plasticity protocol are performed considering various linear and non-linear plausible synaptic memristive dynamics. We consider memristive dynamics bounded by artificial hard conductance values and limited by the natural dynamics evolution toward asymptotic values (soft-boundaries). We quantitatively analyze the impact of resolution and non-linearity properties of the synapses on the network training and classification performance. Finally, we demonstrate that the non-linear synapses with hard boundary values enable higher classification performance and realize the best trade-off between classification accuracy and required training time. With reference to the obtained results, we discuss how memristive devices with non-linear dynamics constitute a technologically convenient solution for the development of on-line SNN training.

## 1. Introduction

Spiking Neural Networks (SNNs) received a renewed wave of interest from a computational point of view as a tool to move the huge overload in data analysis from the cloud to the edge. Indeed, they couple the neural network computing power with spike coding of information, which is considered a valid approach to reduce power requirement for the real-time analysis of unstructured data. This enables the process of *in-situ* decision making of autonomous systems (Indiveri et al., 2013). SNNs are a complementary solution to conventional Neural Networks (NNs), which compute with real-valued numbers and are currently used to remotely analyze the data uploaded to the cloud or at the edge only for inference, without online training (Yu, 2018). Both NNs and SNNs require specific hardware to boost their performance and computing speed. On one side, hardware accelerators of NNs, like graphical processing units and tensor processing units, are now widespread in the market. On the contrary, hardware supporting SNNs are mainly based on research platforms. In both cases, though, the lack of parallelism and separation between storage and computing units is still an issue, for which solutions are under investigation. To this aim, emerging memory devices, compatible with back-end of the production line of CMOS technology, and in particular resistive switching random access memories (RRAM), also named memristive devices, are considered among the best candidates for hardware solutions supporting NNs and SNNs. In particular, the so-called *neuromorphic* systems intend to use memristive devices to update, during training, and store, for inference, the synaptic weights of a network.

Since well-established robust and reliable training algorithms, like the back-propagation of the gradient, is available for NNs, the requirements for memristive devices for NN accelerators have already been determined (Chen et al., 2015; Gokmen and Vlasov, 2016; Sidler et al., 2016; Ambrogio et al., 2018; Fumarola et al., 2018; Moon et al., 2018). It has been shown that the memristive dynamics of the synapses, i.e., the evolution of the memristor conductance driven by train of identical pulses, determines the performance of the network (Chen et al., 2015; Gokmen and Vlasov, 2016; Sidler et al., 2016; Fumarola et al., 2018; La Barbera et al., 2018; Brivio et al., 2019a). In particular, NN accelerators trained through back-propagation require a memristive conductance evolving through many evenly-spaced levels (linear dynamics) (Chen et al., 2015; Gokmen and Vlasov, 2016; Sidler et al., 2016; Fumarola et al., 2018). The same agreement on the required memristive synaptic dynamics in SNN can hardly be reached because various training protocols have been investigated with different results (Brivio et al., 2019a). Currently, SNN training attempts include *on-line* spike-based procedures (Payvand et al., 2018; Brivio et al., 2019a; Donati et al., 2019) and *off-line* conventional training of a non-spiking NN that must be afterwards converted into an equivalent SNN (Diehl et al., 2015, 2016; Sengupta et al., 2019). The former allows exploiting the full potential of memristive devices tuneability to achieve a real-time on-line adaptive operation. Among the spike-based training procedures, supervised learning rules inspired by the back-propagation exist (Urbanczik and Senn, 2014; Müller et al., 2017; Donati et al., 2019), which are seldom investigated for systems including realistic simulations of memristive devices (Nair et al., 2017; Payvand et al., 2018). On the contrary, the literature is extremely rich of reports dealing with networks trained by supervised (Brivio et al., 2019a) and unsupervised versions of the so-called Spike Timing Dependent Plasticity (STDP) (Diehl and Cook, 2015; Garbin et al., 2015; Querlioz et al., 2015; Ambrogio et al., 2016; La Barbera et al., 2018). Few reports indicate that non-linear memristive dynamics may be beneficial for STDP-based SNNs (La Barbera et al., 2018; Brivio et al., 2019a). A comprehensive review about neural networks and spiking neural networks including also memristive devices can be found in Bouvieret al. (2019). In addition, the deployment of all the various emerging technologies for brain-inspired computing is extensively described in Spiga et al. (2020).

In this paper, we aim at moving the first steps toward the optimization of the training of a SNN through system-level simulations as a function of various experimentally-inspired memristive dynamics. Neuron model, training protocol, and architecture are also compatible with a hardware implementation in CMOS technology, as in the silicon chip described in Valentian et al. (2019) and Regev et al. (2020). The investigated memristive dynamics include linear and non-linear evolution bounded within extreme maximum and minimum values, as well as a non-linear evolution asymptotically approaching the boundary values (details are reported below). The response of the network is monitored throughout its training against the classification of hand-written digits from the MNIST dataset (Lecun et al., 1998). We choose this particular task in order to allow a direct comparison with other results reported in the literature for NNs (Chen et al., 2015; Garbin et al., 2015; Ambrogio et al., 2018) and SNNs. (La Barbera et al., 2018; Brivio et al., 2019a) Furthermore, the comparison among the various memristive dynamics is performed in a quantitative manner through the definition of figures of merit that apply to any mathematical formulation for synaptic dynamics. We found that non-linear dynamics bounded within extreme values is the most versatile dynamics, which guarantees the best classification performance and the best compromise between training duration and classification accuracy. This result marks a clear difference with respect to the recent finding related to conventional neural network accelerators trained through the back-propagation algorithm, which, according to a general agreement, require linear synapses (Chen et al., 2015; Gokmen and Vlasov, 2016; Ambrogio et al., 2018; Fumarola et al., 2018).

## 2. Methods

### 2.1. Network Architecture and Training

**Figure 1:**
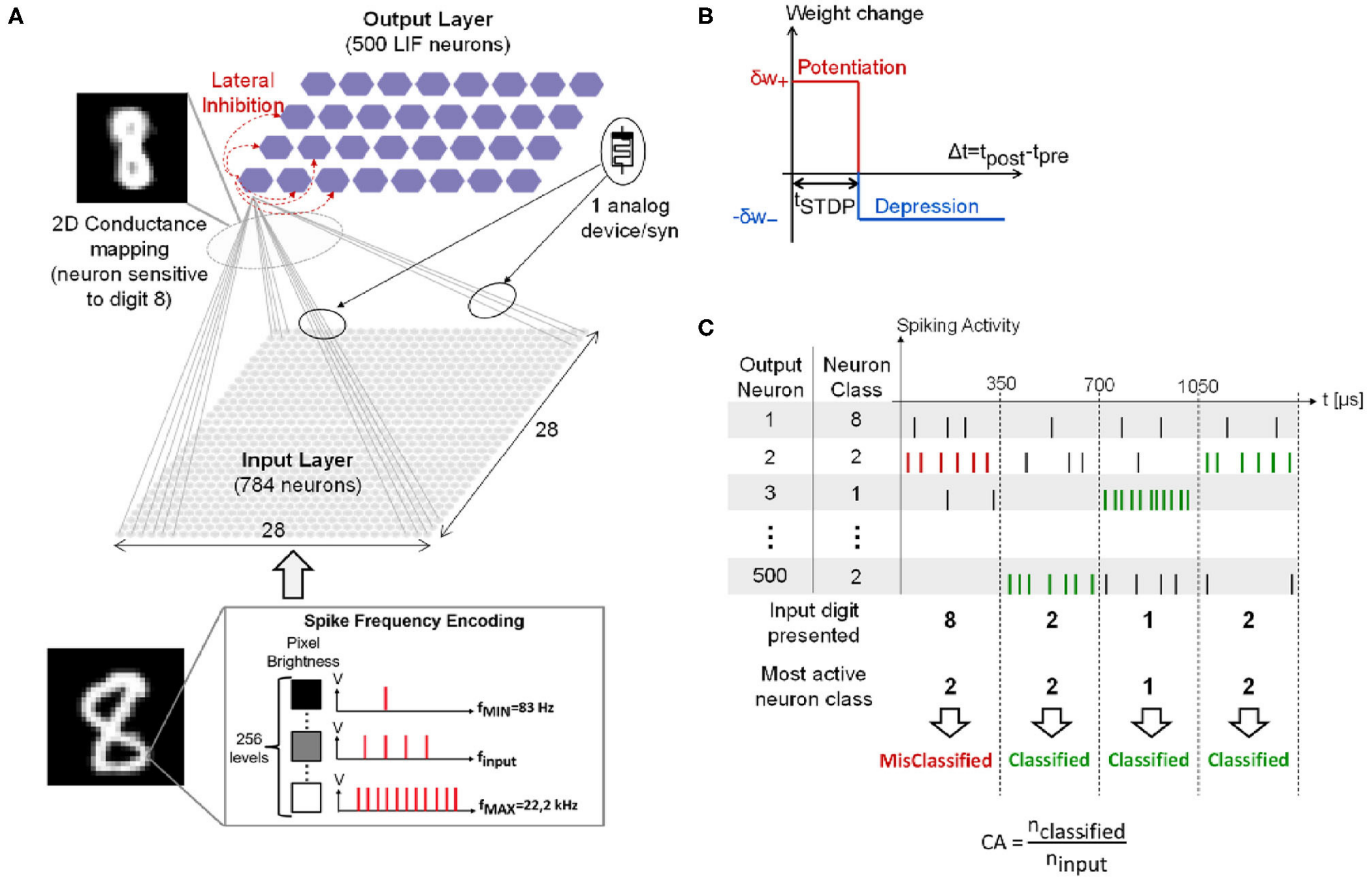
**(A)** Simulated SNN for the MNIST classification. **(B)** Simplified STDP learning rule. **(C)** Example of spiking activity of four output neurons when four different input digits are presented, and Classification Accuracy (*CA*) definition. Adapted with permission from Ly et al. (2018). @IOP Publishing (2018). All rights reserved.

Figure 1A presents the two-layers fully-connected feed-forward SNN simulated for the classification of hand-written digits from the MNIST dataset (Lecun et al., 1998). Simulations are performed with the event-based N2D2 simulator tool (Bichler et al., 2013). The full MNIST dataset is presented only once for training (60,000 training digits), then testing (10,000 testing digits). Each digit is composed of 28 × 28 pixels. The input layer converts the input digit with a spike frequency encoding: each input neuron generates a spike train with a spiking rate f_input_ proportional to the gray level of the corresponding input pixel. f_input_ ranges from f_MIN_ = 83 Hz to f_MAX_ = 22.2 kHz with a total of 256 different gray levels. Spike trains are generated according to a Normal distribution. Each input digit is presented to the network for 350 μs during the training phase. The input layer is composed of 28 × 28 input neurons fully-connected by weighted synapses to the output layer composed of 500 Leaky Integrate-and-Fire (LIF) output neurons with a leak time constant τ_leak_ = 120.0 μs. Note that after an output neuron fires a spike, it cannot integrate any incoming spikes for a refractory period t_refrac_ = 1 ns. It also prevents all the other neurons of the layer from integrating incoming spikes for a period t_inhibit_ = 10 μs, referred to as *lateral inhibition*. This allows implementing a Winner-Take-All (WTA) network between all the neurons (Bichler et al., 2013). In addition, a slight delay in the firing time of output neurons has been introduced: when an output neuron reaches its threshold value, it fires a spike after a delay t_emit_. The parameter t_emit_ for each output neuron has been randomly drawn from a normal distribution with a mean value μ = 0.1 ns and a standard deviation σ = 1 ps. This facilitates the implementation of the WTA process. These parameters have been optimized by a genetic algorithm. The network is trained with an unsupervised simplified Spike-Timing-Dependent Plasticity (STDP) rule (Figure 1B) (Suri et al., 2011; Querlioz et al., 2015): if the post-synaptic neuron spikes after the pre-synaptic neuron within a STDP time window t_STDP_ = 60.0 μs, the synapse increases its synaptic weight by a quantity δw_+_ (synaptic *potentiation* event). Otherwise, its synaptic weight is decreased by a quantity δw_−_ (synaptic *depression* event). Quantities δw_+_ and δw_−_ follow different dynamics models described in the following section. The weights are bounded between [0, 1] and are initialized to the value of 0.8 before training. From a hardware point of view, the initialization of devices to a predefined value is more straightforward than a random initialization. In particular, the weight value of 0.8 (i.e., high memristive conductance) is coherent with an initialization performed in hardware with only an electroforming step, which is required for a large class of memristive devices (Brivio and Menzel, 2020). Furthermore, the initialization does not influence the obtained classification performances as demonstrated in Querlioz et al. (2013).

During the training phase, each output neuron becomes sensitive to a specific class of digit as illustrated in the 2D conductance mapping in the top left of Figure 1A (class of digit “8”). After training, each output neuron is associated with the digit it is the most sensitive to. This represents the class of the neuron. To assess network performance during the testing phase, the Classification Accuracy (*CA*) is computed as defined in Figure 1C. Each input digit is presented to the network for 350 μs and the output neuron that spikes the most within this time window—the most active neuron—corresponds to the network response. If the most active neuron class coincides with the input digit, the digit is successfully classified (green spikes). Otherwise, the digit is misclassified (red spikes). The *CA* is calculated as the ratio between the number of successfully classified digits, n_classified_, and the number of input digits, n_input_ (bottom of Figure 1C). As there are multiple ways to hand-write the same digit, increasing the number of output neurons allows for an improvement of network performance as demonstrated in Querlioz et al. (2015). Indeed, this enables the network to have at its disposal several neurons specialized to different hand-writings of the same digit. As shown in Querlioz et al. (2015), the increase of *CA* with the number of output neurons saturates after 500 output neurons.

It is worth pointing out that the network architecture and operation are implemented according to the real hardware possibilities of the current CMOS and memristive technologies. In particular, contrary to Querlioz et al. (2015) who implemented the same network as the present one, homeostasis, which, e.g., adjusts each individual output neuron threshold on the basis on its instantaneous firing rate, is not included. As a matter of fact, Querlioz et al. (2015) shows that homeostasis can improve the classification accuracy by about 10%. On the other hand, the hardware implementation of homeostasis would require memory banks to store each individual neuron threshold values and one additional capacitor per neuron to probe each neuron firing rate, which will have a prohibitive impact on the required silicon real estate (Dalgaty et al., 2019). Some pioneering works are trying to address this issue with the help of memristive technology (Dalgaty et al., 2019), but a hardware-compatible homeostatic process over a large number of neurons has not been elaborated yet.

Furthermore, the classification scheme can also be improved with a voting procedure that takes into account the average firing rate of each neuron pool as in Diehl and Cook (2015), instead of considering only the individual neuron that fires the most as in the present implementation. However, the voting procedure based on the individual neuron firing rate eases the circuit complexity and is only marginally influencing the network performances. Indeed, Querlioz et al. (2015) obtained a classification accuracy (94.5%) very close to that of Diehl et al. (95%).

### 2.2. Models for Memristive Dynamics

**Figure 2:**
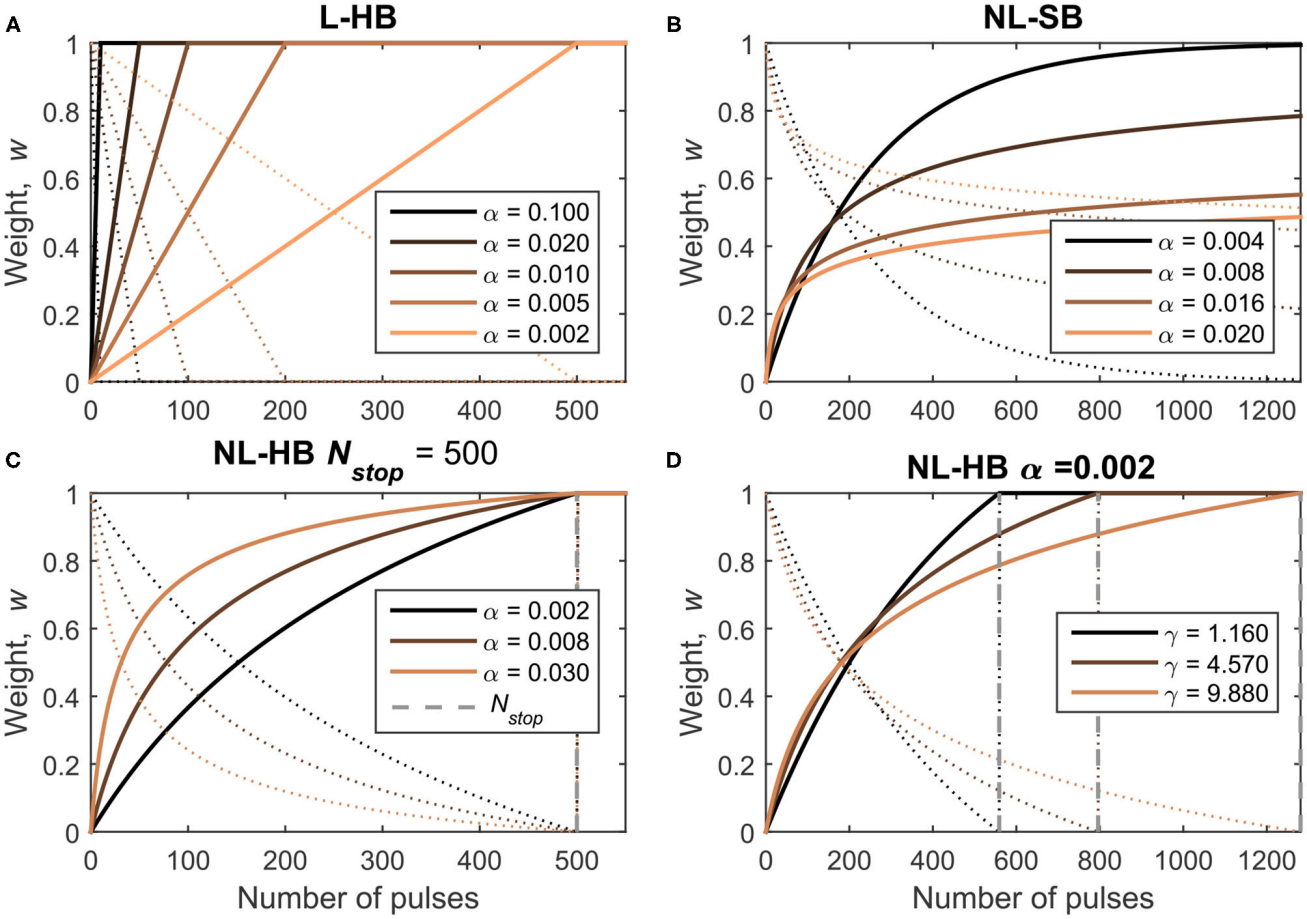
Investigated dynamics: **(A)** Linear Hard-Bound (L-HB); **(B)** Non-Linear Soft-Bound (NL-SB) and **(C,D)** Non-Linear Hard-Bound (NL-HB). Dynamics are plotted as a function of the number of pulses for potentiation and depression operations (straight and dotted lines, respectively). The various dynamics cases are defined in Table 1.

The synaptic dynamics corresponds to the evolution of the weight of an artificial synapse (proportional to the memristive device conductance) when subjected to a train of identical pulses. Considering bipolar memristive synapses, trains of pulses of a given voltage polarity can lead to weight potentiation and trains of pulses with the opposite polarity lead to weight depression. As evident from the recent literature (Fumarola et al., 2018) and pointed out by part of the present authors in Frascaroli et al. (2018), the more general memristive conductance dynamics usually follows a non-linear evolution with a slow approach to the maximum and minimum values. Such dynamics can be described by a *non-linear soft-bound* (NL-SB) model, which has a particular importance in the field of computational neuroscience. Indeed, Fusi and Abbott (2007) demonstrated that NL-SB synapses generally endow a SNN with a larger memory capacity (capacity of storage of memories) compared to synapses whose weight evolve linearly between two boundary values. This latter synaptic model will be referred to as *linear hard-bound* (L-HB) synapses in the following. Fusi and Abbott (2007) showed that L-HB synapses perform better than NL-SB ones only in the particular case of a balanced network, i.e., a network in which the rate of potentiation is the same as the rate of depression events. From an experimental point of view, a memristive dynamics is usually approximated with a L-HB dynamics by interrupting a NL-SB one after a certain number of pulses at the cost of reduced conductance window, ${\;}^{G_{max}}{/}_{G_{min}}$. Examples of experimental reports can be found in Jang et al. (2015), Wang et al. (2016), and Bousoulas et al. (2017). A third generic case, which we will call *non-linear hard-bound* (NL-HB), consists in a non-linear dynamics interrupted at arbitrary boundary values. The boundary values are strictly reached after a certain finite number of consecutive weight increase or decrease events. As already mentioned, the NL-SB case is different because the weight boundaries are reached as asymptotic values after an infinite number of pulses (from a experimental point of view, tests up to few thousand pulses have been performed; Brivio et al., 2019a). The investigated L-HB and NL-SB dynamics in potentiation (conductance increase) and depression (conductance decrease) are shown in Figures 2A,B as solid and dotted lines, respectively. Figures 2C,D report various investigated NL-HB cases, for potentiation and depression operations as solid and dotted lines, respectively. The examples reported in Figure 2 correspond to specific mathematical expressions and parameterizations of the dynamics models as described in the following.

Formally, the weight dynamics can be expressed in a differential form in the continuous domain as a variation of the weight, *dw*, per pulse, *dn*. The weights are always positive because they are represented by the conductance value of a physical device. Furthermore, it must be pointed out that hard-bound cases are experimentally obtained by interrupting a generic soft-bound dynamics, which therefore reduces the conductance window of hard-bound cases. Despite this fact, all the dynamics cases are simulated with the same conductance window considering the weight as the normalized version of the conductance between [0, 1], as plotted in Figure 2. Therefore, for all the following equations one should consider *w* ∈ [0, 1] and $\frac{dw_{\pm }}{dn}=0$ outside the interval [0, 1]. In particular, the L-HB dynamics is given by

(1) $$\begin{array}{r} \frac{dw_{\pm }}{dn}=\pm \alpha_{\pm }, \end{array}$$

with α_±_ ∈ (0, 1] and where the (·)_+_ and the (·)_−_ stand for potentiation and depression, respectively. Following Fusi and Abbott (2007), Frascaroli et al. (2018), and Brivio et al. (2019a), the NL-SB equation is given by

(2) $$\left\{ \begin{array}{l} \frac{dw_{+}}{dn}=\alpha_{+}{\left(1-w\right)}^{\gamma_{+}}\\ \frac{dw_{-}}{dn}=-\alpha_{-}w^{\gamma_{-}} \end{array}\right.$$

with α_±_ ∈ (0, 1] and γ_±_≥1. It is evident from Equation (2) that the weight variation tends to nullify as *w* approaches the boundary values. The NL-HB dynamics is the truncated version of the NL-SB properly re-scaled between 0 and 1, as follows

(3) $$\{\begin{array}{l} \frac{dw_{+}}{dn}=\frac{\alpha_{+}}{w_{stop,+}}{\left(1-w\;\cdot \;w_{stop,+}\right)}^{\gamma_{+}}\\ \frac{dw_{-}}{dn}=-\frac{\alpha_{-}}{w_{stop,-}}{\left(w\cdot w_{stop,-}+1-w_{stop,-}\right)}^{\gamma_{-}} \end{array},$$

where α_±_ ∈ (0, 1], γ_±_ ∈ [1, +∞). *N*_*stop*, ±_ are the values of *n* at which the corresponding NL-SB dynamics is truncated to get a NL-HB one. *w*_*stop*, ±_ are the normalization terms that depend on the value of *N*_*stop*, ±_, as shown in the Supplementary Material.

**Table 1:** List of the investigated dynamics defined by the values of their parameters α, γ, and *N*_*stop*_.

	**α_±_**	**γ_±_**	**N_***stop*, ±**_**	**η**	**λ**
L-HB	0.1	–	–	10	0
	0.02	–	–	50	0
	0.01	–	–	100	0
	0.005	–	–	200	0
	0.002	–	–	500	0
NL-SB	0.02	9	–	500	0.02
	0.016	7	–	500	0.020
	0.008	3	–	500	0.010
	0.004	1	–	500	0.005
NL-HB	0.002	3	500	402	0.006
	0.008	3	500	225	0.015
	0.03	3	500	90	0.047
	0.002	1.16	559	500	0.004
	0.002	4.57	796	500	0.006
	0.002	9.88	1281	500	0.009

*The corresponding values for resolution, η, and non-linearity, λ, are also reported*.

It is worth making some additional clarifications. Each dynamics case is described by one or more free parameters which are chosen as described in the following. It is clear from Equations (1) and (2) that α_±_ is the step height when departing from the boundary value for the L-HB and the NL-SB dynamics. Indeed, the weight moves away from the lower boundary value, *w* = 0 for potentiation (resp. higher boundary value, *w* = 1 for depression) with a weight change equal to α_+_ (resp. −α_−_). For the NL-HB case, the first step height is ${\;}^{\alpha_{\pm }}{/}_{w_{stop,\pm }}$. In addition, the weight change step is constant throughout the entire weight range for the L-HB case; it decreases from α to 0 for the NL-SB case; and it decreases from ${\;}^{\alpha_{\pm }}{/}_{w_{stop,\pm }}$ to a finite value greater than 0 for the NL-HB case. The parameter γ_±_ introduces an additional non-linearity factor, whose effect can be appreciated from Figure 2B. For each dynamics case, potentiation and depression evolution are considered identical, i.e., with the same values of the free parameters, α_+_ = α_−_, γ_+_ = γ_−_, and *N*_*stop*, +_ = *N*_*stop*, −_. As a consequence, the pace of approaching and departing to and from a given weight value is the same only for linear synapses. On the contrary, non-linear synapses are characterized by a certain asymmetry between potentiation and depression. For instance, a NL-SB synapse can be potentiated with a significant rate away from a weight value close to 0 (*w*≈0). In turn, at the same value, the depression rate is close to 0 because ${\;}^{dw_{-}}{/}_{dn}=-\alpha_{-}w^{\gamma_{-}}\approx 0$. As a matter of fact, the asymmetry between potentiation and depression dynamics is usually present in real devices (Lee et al., 2015; Frascaroli et al., 2018). The impact of asymmetry between potentiation and depression dynamics on the performances of a neuromorphic system has been investigated in some detail for networks trained through back-propagation of the error (Chen et al., 2015; Agarwal et al., 2016; Fumarola et al., 2018) and only partially in spiking networks (La Barbera et al., 2018). However, a procedure to decouple the effect of asymmetry from that of non-linearity has not been proposed yet.

The memristive evolution in the network is determined by the STDP rule described in the previous section and the Equations (1)–(3). In fact, when the pre- and post-spikes are emitted according to the potentiation (depression) window in Figure 1B, a potentiation (depression) pulse is delivered to the memristive synapse driving a weight change equal to δ*w*_+_ (δ*w*_−_). The quantity δ*w*_±_ is determined by the current synaptic weight *w* and by the dynamics parameters in Table 1 according to Equations (1)–(3). The programming of memristive device with CMOS neuron circuit, in STDP-based schemes, has been investigated in a number of works, which highlighted the need to include compact interface electronics. Pedretti et al. (2017) demonstrated STDP protocols on real 1 transistor-1 memristor structures based on the temporal overlapping on pre- and post-synaptic pulses driven by microcontrollers. Mostafa et al. (2016) designed a memristor/CMOS neuron interface constituted by 4 CMOS transistors to drive weight depression and potentiation operations separately within the framework of a generalized version of STDP. Covi et al. (2018) tested the same structure by wire-connecting a single memristor to two 350 nm technology CMOS neurons. The neurons delivered the correct programming pulses to obtain both analoge and digital memristive responses. In Brivio et al. (2019a), a 6 transistor-1 memristor structure is proposed to control synaptic potentiation, depression, and read operations in an implementation of a generalized version of STDP. The system-level simulation implemented in the present work are compatible with such implementation details.

The main weighting property of a synapse is its resolution, i.e., the number of weight values that it can store. The resolution of a synapse has a direct impact on the performances of a network (Bill et al., 2014; Brivio et al., 2019a). However, while the definition of number of levels is straightforward for L-HB dynamics, the same does not hold true in the case of non-linear weight evolution, because in this case the weight values are not evenly spaced. As a matter of principle, for NL-HB case, the resolution could be evaluated equal to the number of update events that are necessary to bring the weight from one boundary value (e.g., 0) to the opposite (i.e., 1), i.e., exactly *N*_*stop*_. However, this is not a proper definition because the weight can be driven from 0 to 1 with the same number of steps but through various and very different trails. In particular, Figure 2C reports three NL-HB cases for which the same number of pulses is required to cover the full weight range but show very different dynamics. It is reasonable to associate different resolutions (or effective number of levels) to the three examples. In addition, it is worth pointing out that the number of pulses required to cover the full weight range is not a good definition for the NL-SB, which strictly requires an infinite number of steps to reach the boundary values. Indeed, the non-linear cases reported in Figure 2 should be associated to different resolutions from a purely mathematical point of view. These considerations are completely independent from the effect of the noise and variability that unavoidably affect any real memristive device (Yu et al., 2013; Frascaroli et al., 2015, 2018; Covi et al., 2016; Brivio et al., 2017, 2019b). The impact of noise and variability has been investigated for some specific networks and some applications, demonstrating a general tolerance of neuromorphic systems against memristive synapse variability and noise (Querlioz et al., 2013; Garbin et al., 2014; Burr et al., 2015; Covi et al., 2016; Bocquet et al., 2018). Since we want to restrict the present study to a purely theoretical basis on the very impact of synaptic dynamics on network performances, the effect of noise and variability are left to a future work.

For the reasons above, we arbitrarily define an estimator for the resolution (effective number of levels) of the memristive device which can be applied to any generic dynamics expressed as a weight variation (*dw*) per pulse (*dn*) in the continuous domain, $\frac{dw_{\pm }}{dn}=f_{\pm }\left(w\right)$ [*f*_±_(*w*) must be differentiable for *w* ∈ (0, 1)]. The resolution, η, is defined as

(4) $$\eta ={\left\{{\int_{0}^{+\infty }\left[\frac{dw}{dn}\right]}^{2}dn\right\}}^{-1}.$$

Equation (4) returns the correct number of levels for the trivial L-HB case, i.e., equal to the number of pulses to go from one boundary to the other one, and a reasonable estimate for the non-linear cases, as discussed in the Supplementary Material. η assumes analytical expressions for the dynamics cases under study, as reported in the Supplementary Material. It is just worth noticing that $\eta {=}^{1}{/}_{\alpha_{\pm }}$ for the L-HB case and is proportional to ${\;}^{1}{/}_{\alpha_{\pm }}$ for the NL-SB (in agreement with Fusi and Abbott, 2007) and the NL-HB cases.

According to the discussion above and to the recent literature, a second property of weight dynamics is its non-linearity (λ), which can be defined as the average curvature of the weight evolution as a function of the number of potentiation or depression pulses, *w*(*n*):

(5) $$\lambda =\frac{4}{\pi }\int_{0}^{+\infty }\frac{\left|w^{\prime \prime }(n)\right|}{{\left\{1+{\left[w^{\prime }(n)\right]}^{2}\right\}}^{3{/}_{2}}}dn,$$

where (·)′ and (·)″ indicate the first and the second derivative with respect to *n*.

In this work, we investigate the impact of these synaptic properties, namely the resolution, η, and the non-linearity, λ, on the training and performance of the SNN described above. In particular, we consider various L-HB, NL-SB, and NL-HB dynamics, as reported in the Table 1 and shown in Figure 2. Note that the L-HB cases are only characterized by different values of the resolution, because only one free parameter exists. For the NL-SB case, it is possible to investigate different dynamics for the same resolution, i.e., with different non-linearities. NL-HB cases are chosen in a way to have the same *N*_*stop*, ±_ (cases 1–3) or the same resolution, η (cases 4–6). We investigate resolution values up to 500 because this is the one that guarantees good performance on MNIST classification on the linear case, according to previous literature results (La Barbera et al., 2018; Brivio et al., 2019a) and as it will be evident also in the following. The free parameters of the various dynamics cases are generally compatible with experimental data as in Frascaroli et al. (2018) and Brivio and Menzel (2020). For the sake of completeness, it is worth noticing that dynamics of a memristive device depends on the properties of the constitutive materials and on the programming conditions. For instance, memristors featuring double insulating layers have been reported to show more gradual conductance evolution than devices with a single insulating material (Park et al., 2016; Wang et al., 2016; Moon et al., 2018; Brivio and Menzel, 2020). The response speed might depend on the diffusivity of the mobile ionic species as well. It is a property of the insulating materials itself, which can also be slightly tuned by doping, strain, or by changing the atomic structure and porosity (Azghadi et al., 2020; Brivio and Menzel, 2020). Furthermore, the programming scheme influences the dynamics. Indeed, strong programming conditions (high voltage or long pulses) result in large conductance changes with a few pulses (Frascaroli et al., 2018). It is worth specifying that all the parameters (α, γ, *N*_*stop*_) defining the memristive dynamics affect both resolution and non-linearity at the same time. More details can be found in the Supplementary Material.

## 3. Results

**Figure 3:**
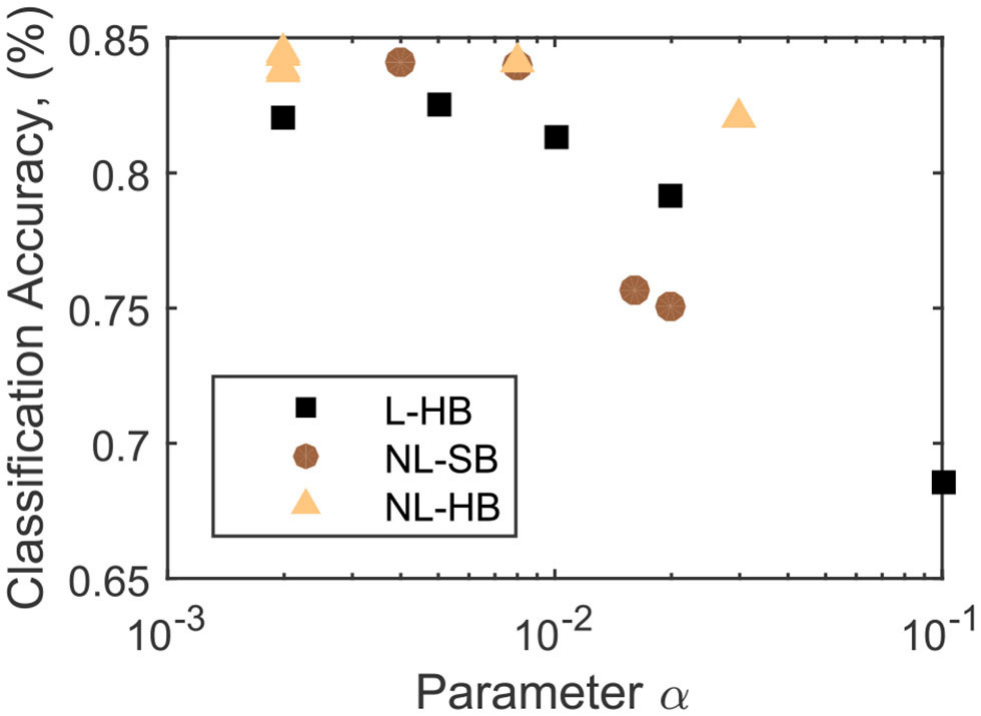
Classification accuracy as a function of the parameter α for the L-HB, NL-SB, and NL-HB cases.

As discussed in the previous paragraph, the mathematical formulation of all the investigated dynamics comprises a parameter α, the only parameter that is present in all investigated synaptic dynamics. In Figure 3, the classification accuracy, *CA*, is shown to decrease as a function of α for the investigated cases. This observation is expected because ${\;}^{1}{/}_{\alpha }$ is equal to the synaptic resolution of the L-HB dynamics and is proportional to that of the non-linear ones. It is already evident that the non-linear cases perform better than the linear ones for a wide range of the parameter α, in agreement with previous publications (La Barbera et al., 2018; Brivio et al., 2019a). However, only the evolution as a function of α does not catch the entire complexity of the problem because, for the NL-SB and NL-HB cases, α affects both resolution and non-linearity. Indeed, the various types of weight dynamics in Figure 3 follow a different decreasing trend. The maximum reached classification accuracy settles close to 85%, which is lower than the best results on theoretical SNNs (Diehl and Cook, 2015). However, as stated above, the aim of the present work is to test SNNs constituents and architectures that can be possibly realized in hybrid CMOS/memristor technology (Valentian et al., 2019; Regev et al., 2020). As discussed in the Methods section, the inclusion of a homeostatic rule, which is of difficult hardware implementation, would recover a classification accuracy close to the best state of the art results, as demonstrated by Querlioz et al. (2015) with the same network as the one implemented in this work. The values of the collected classification accuracy are reported in the Supplementary Material.

**Figure 4:**
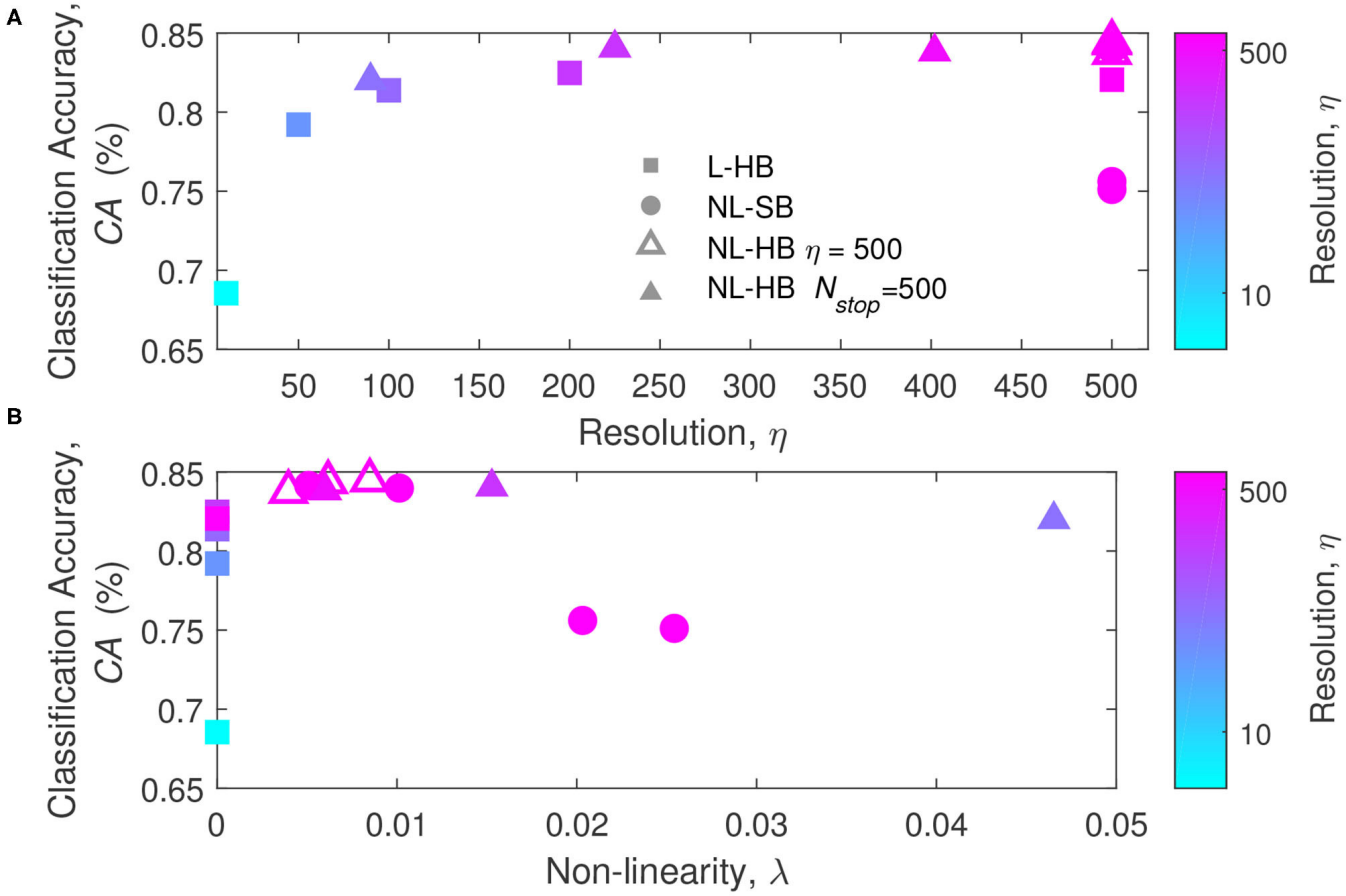
Classification accuracy as a function of the parameters η **(A)** and λ **(B)**, for the L-HB, NL-SB, and NL-HB cases. The symbols color follow the resolution value, η, according to the color bars reported on the right sides of the panels.

To get a deeper insight on the factors affecting the network performances, the classification accuracy is plotted as a function of η and λ in Figure 4, which reports the first of the main results of the paper taking advantage of the mathematical toolkit described in the previous section. Figure 4A shows that there is a general trend of increasing *CA* with the synaptic resolution, η. Different resolution values are shown for the dynamics with hard-bounds, linear (squares) and non-linear (triangles), i.e., L-HB and NL-HB. They show a very similar trend with slightly higher *CA* for the non-linear case. The investigated NL-SB dynamics (circles) share the same resolution (η = 500) but they give different *CA* results. In particular, for the NL-SB, the *CA* is reduced by the increase of non-linearity, λ, as shown in Figure 4B. In turn, the non-linearity does not affect significantly the performances of the non-linear synapses with hard bounds, for which the *CA* remains almost stable over a wide range of λ values. It is important to point out that NL-HB synapses with the highest non-linearity λ = 0.047 are also characterized by the lowest resolution η = 90 (please consider that the symbol color is indicative of the synaptic resolution η, according to the color reported on the right-hand side of the figure). In any case, both the low resolution and the high non-linearity affect the classification performance only by a small amount. In addition, it is worthless to notice that the L-HB synapses are all described by zero non-linearity (λ = 0).

**Figure 5:**
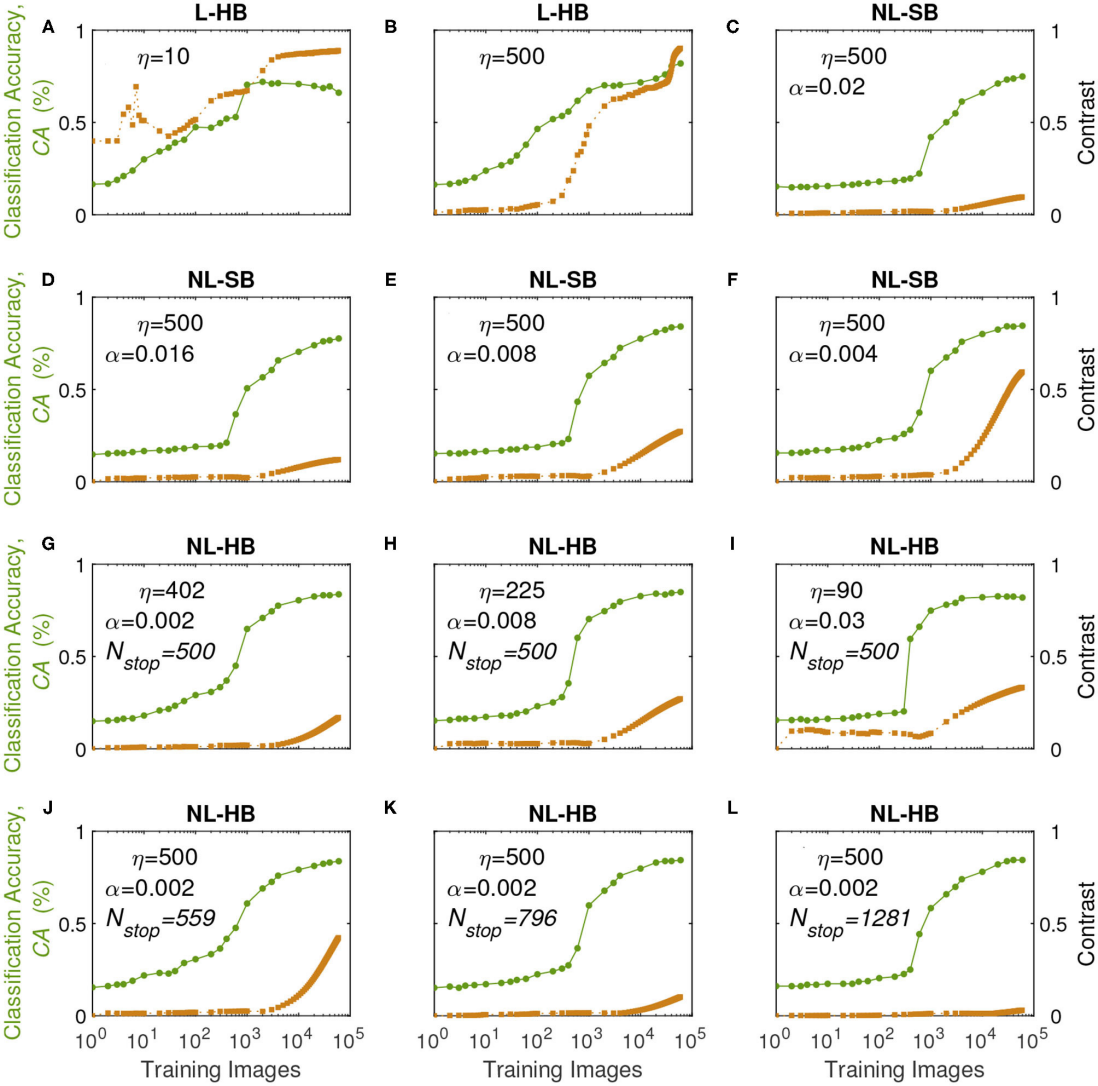
Classification Accuracy (*CA*, left axis) and weight contrast (right axis, as defined in the main text) as a function of the number of training images presented to the SNN for various dynamics in the different panels.

**Figure 6:**
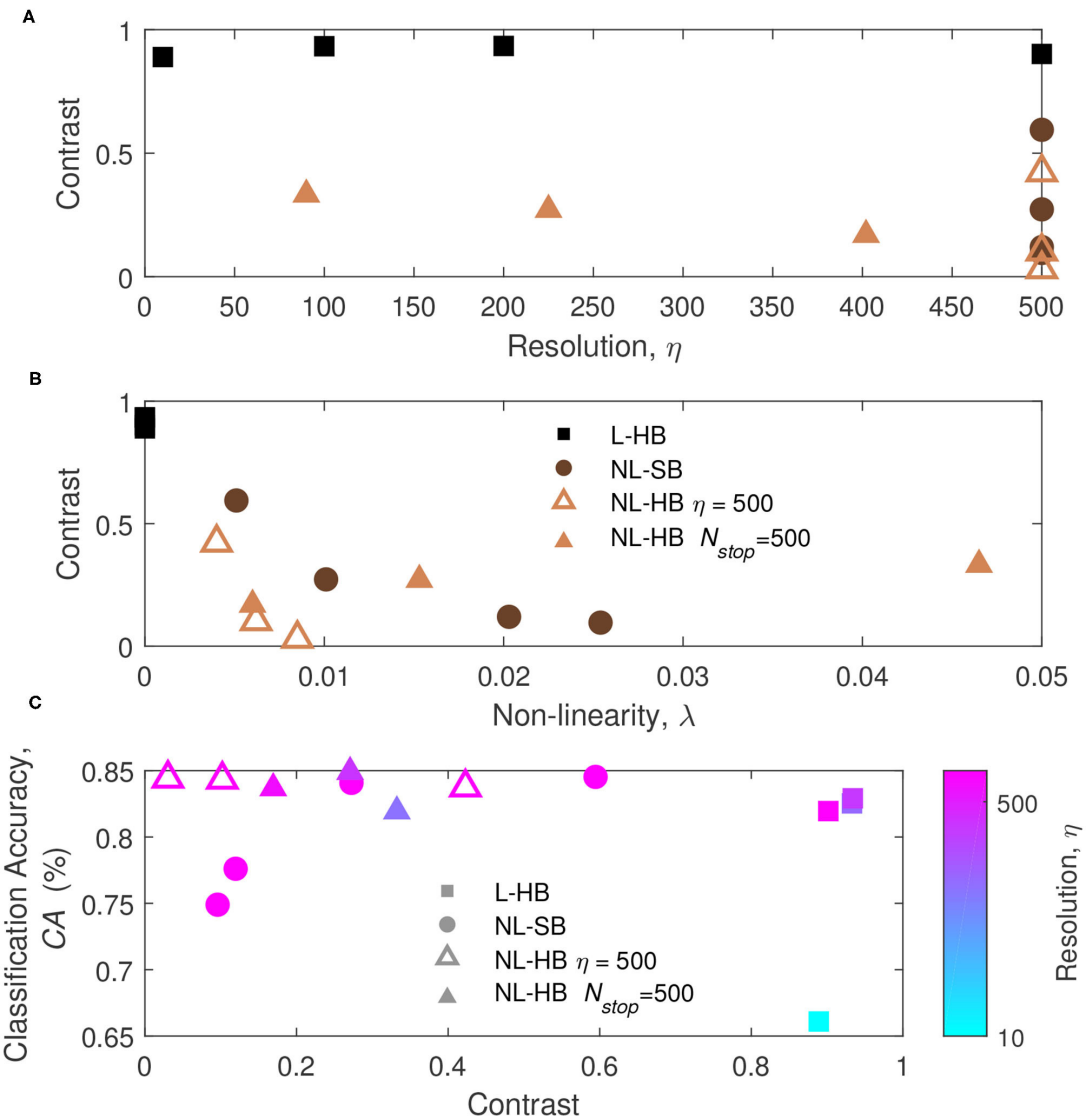
**(A,B)** Weight contrast at the end of the training as a function of the parameters η and λ, for the L-HB, NL-SB, and NL-HB cases. **(C)** Classification accuracy, *CA*, as a function of the weight contrast. The symbols color follow the resolution value, η, according to the color bar reported on the right side of panel.

In order to understand the previous results we monitor the learning dynamics, i.e., the *CA* as a function of the training time (i.e., number of training digits), which usually displays a growth and a saturation toward the final maximum value. The learning dynamics for all the investigated synaptic models are reported in Figure 5 (circles, left axis). With training, the synaptic weights evolve in a way that enables the distinction between the digits. In particular, it is well known that linear synapses, i.e., characterized by weight-independent plasticity, tend to develop bi-modal weight distributions after training (Song et al., 2000; van Rossum et al., 2000; Rubin et al., 2001; Billings and van Rossum, 2009). In this case, the weight values accumulate at the edges of the useful weight range, i.e., [0,1] in the present case. On the contrary, non-linear synapses with weight-dependent plasticity tend to result in a uni-modal weight distribution, with weight values accumulating in a value somewhere in the middle of the allowed weight range (Song et al., 2000; van Rossum et al., 2000; Rubin et al., 2001; Morrison et al., 2008; Billings and van Rossum, 2009; Brivio et al., 2019a). This is the result of the fact that strong (weak) synapses with non-linear dynamics are weakly potentiated (weakly depressed), which was shown to improve the memory capacity of the network on one side and, on the other, limit the synaptic specialization of the classification layer (Fusi and Abbott, 2007; Brivio et al., 2019a). As a matter of fact, in general, weight-dependent synapses and uni-modal distributions are considered less informative (Hennequin et al., 2010), because they correspond to a lower degree of specialization than weight-independent synapses and bi-modal distributions. Conversely, uni-modal distributions are considered more biologically realistic (Morrison et al., 2008). The weight distributions of the investigated cases at the end of the training are reported for the sake of completeness in the Supplementary Figure 2. In order to monitor the development of a weight specialization that enables the network to classify the input images, we analyze the clustering of the weights into two groups as a function of training through the *k-means* algorithm and consider the distance between the centers of the two clusters as a measure of the network specialization, which we will call weight contrast. Indeed, intuitively the weight contrast can be considered as the ability to take advantage of a wide portion of the available weight range. Other methods to group the weight values into two clusters are analyzed in the Supplementary Material and are in agreement with the *k-means* algorithm results. The weight contrast is reported in Figure 5 (squares, right axis) for the various dynamics cases. It is possible to notice that the linear cases develop a large weight contrast at the end of training (Figures 5A,B) in agreement with the general discussion above. The non-linear cases show lower weight contrast than the linear cases but with significant variations depending on the dynamics parameters (for instance, cf. Figures 5J,L for two different NL-HB cases). The weight contrast at the end of the training is plotted against the parameters η and λ in Figures 6A,B, respectively. Figure 6A shows that the L-HB case results in about the same contrast for every resolution, while in the NL-SB case the same synaptic resolution can give very different weight contrasts, depending on the non-linearity, λ (Figure 6B). The NL-HB case is the most interesting, because the additional parameter *N*_*stop*_ allows to increase the contrast either by reducing the resolution, as shown by the filled triangles in Figure 6A, or by reducing the non-linearity at equal resolution, as shown by the empty triangles in Figure 6B. Finally, Figure 6C reports the *CA* as a function of the weight contrast. It shows that L-HB synapses (squares) are all characterized by high contrast but only those with high resolution achieve high classification accuracy (please notice, again, that the symbol color is indicative of the synaptic resolution η, according to the color reported on the right-side of the figure). NL-SB synapses (circles) achieve high *CA* only when the weight dynamics develops a high contrast. This is obtained by reducing the non-linearity (please compare with Figure 6B). The classification results of the NL-HB synapses (triangles) are almost independent from the weight contrast obtained at the end of the training.

The results of Figures 4, 6 constitute already a relevant result with respect to the state of the art. Indeed, linear synaptic dynamics is often considered as the best solution for any kind of hardware neural network, so that large efforts are spent to improve linearity of memristor dynamics (Wang et al., 2016; Bousoulas et al., 2017; Chen et al., 2019). Such belief may have raised as a generalization of the results of exemplary works on NN accelerators trained by back propagation of the error generalized to other networks and other training protocols (Burr et al., 2015; Fumarola et al., 2018). As a matter of fact, as mentioned above and shown in Figure 6B, linearity improves weight contrast and sustains the specialization of the network. However, it has been demonstrated that non-linear synapses improve memory lifetime and memory capacity of a network in which the rates of potentiation and depression events are not perfectly balanced (Fusi and Abbott, 2007). Furthermore, van Rossum et al. (2000) pointed out that STDP tends to make potentiated synapses more and more potentiated. Indeed, as a synapse is strengthened, its correlation with the post-synaptic neurons increases thus leading to a further potentiation. Van Rossum et al. demonstrated that this destabilizing tendency of STDP can be profitably counterbalanced by introducing weight-dependent plasticity (i.e., a non-linear dynamics) which produces a certain competition among synapses. The results in Figures 4, 6 can be generically ascribed to a different balance between contrast decrease, increase of memory lifetime, and synaptic competition with increasing non-linearity.

This result marks a difference with respect to memristor-based neural network accelerators trained by global error back-propagation for which the achievement of high weight contrast and bi-modal weight distribution taking advantage of the full weight range is fundamental for a successful training (Sidler et al., 2016; Fumarola et al., 2018).

Another important aspect to consider is the duration of the training process, which for some applications must be reduced to a minimum. To evaluate it, we define the parameter Δ_*train*_ as the fraction of training images required to reach 99% of the final classification accuracy over the total number of digits available for training, *n*_*tot*_ (with *n*_*tot*_ = 60,000 here). In symbols,

(6) $$\begin{array}{r} \Delta_{train}=\frac{n_{train}\left(CA=99\%CA_{max}\right)}{n_{tot}}, \end{array}$$

**Figure 7:**
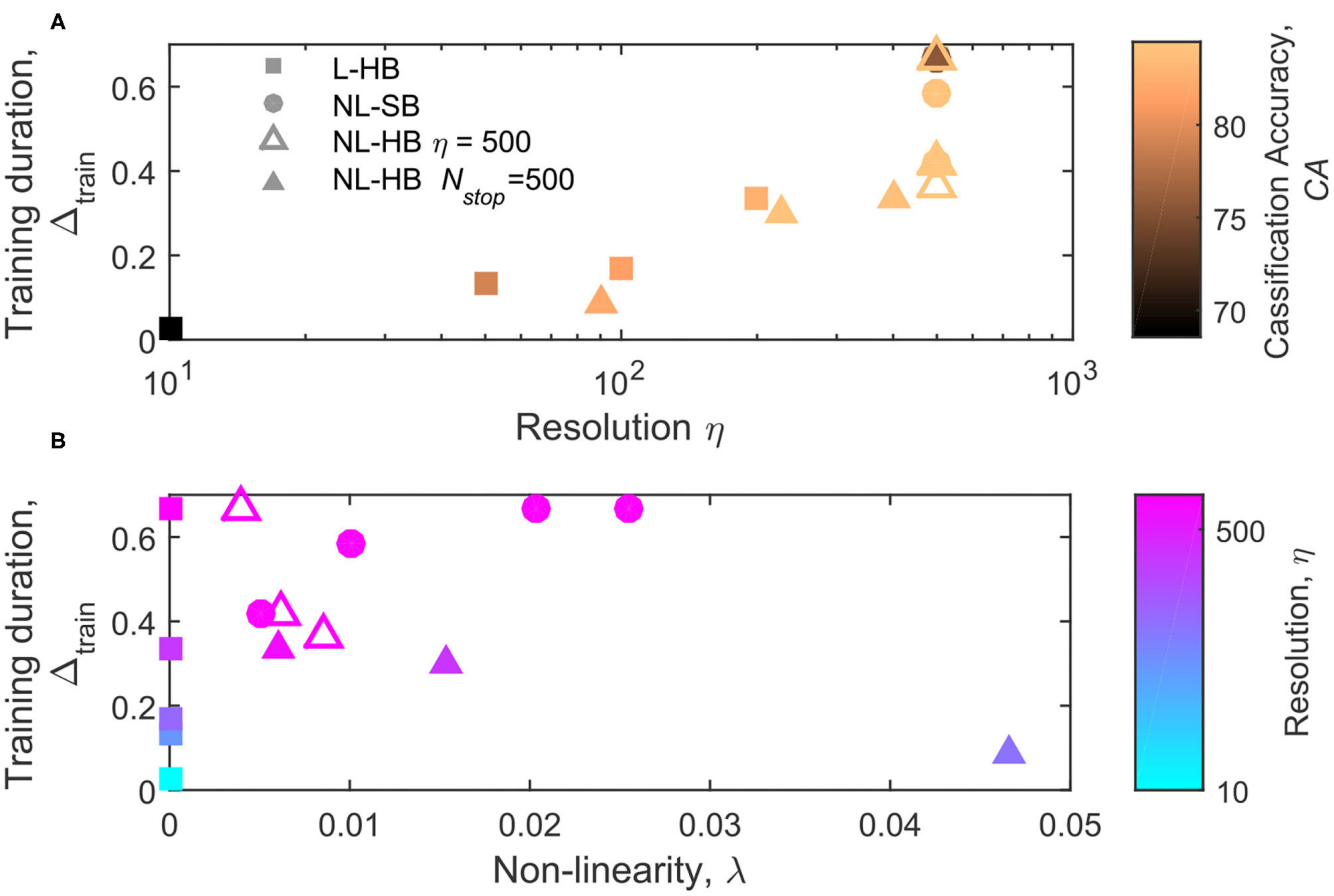
Training duration, *N*_*train*_, as a function of the parameter η **(A)** and λ **(B)**, for the L-HB, NL-SB, and NL-HB cases. The symbols colors in follow the classification accuracy, CA, in **(A)** and the resolution value, η, in **(B)** according to the color bars reported on the right sides of the panels.

The parameter Δ_*train*_ is shown as a function of η and λ in Figures 7A,B, respectively. Figure 7A indicates a correlation between the synapse resolution and Δ_*train*_. The correlation is somehow expected in case of a strong tendency to the formation of a bi-modal weight distribution, i.e., linear synapses (squares). Indeed, if the weight values tend to concentrate at the boundary values, the number of steps required to move the weight values from a generic initial one to the boundary scales with the synapse resolution. In agreement with this interpretation, the correlation between Δ_*train*_ and η is not perfect for the non-linear cases, because for the same resolution very different Δ_*train*_ values are obtained, as shown in Figure 7A in particular for the NL-SB cases (filled circles). Interestingly, the evolution of Δ_*train*_ as a function of λ follows opposite trends for soft and hard bound cases (also considering only the points at equal resolution, empty triangles and filled circles), as visible in Figure 7B. It is worth noticing that NL-SB and NL-HB with 500 levels resolution also show the same evolution of contrast as a function of non-linearity, as shown by filled circles and empty triangles in Figure 6B. Therefore, the opposite trends of Δ_*train*_ as a function of non-linearity cannot be explained by the need to develop, during training, a weight contrast that scales differently with non-linearity for NL-SB and NL-HB dynamics. On the contrary, the classification accuracy of NL-SB and NL-HB dynamics with the same 500 levels resolution follows opposite trends as a function of non-linearity, as indicated in Figure 4B (though the change for the NL-HB case is very modest). Therefore, the fact that both accuracy and training time follow opposite trends as a function of non-linearity can be an indication that, for non-linear dynamics, the training time is influenced by the maximum classification accuracy allowed by the particular synaptic dynamics. Finally, also considering the training time, the NL-HB cases (triangles) demonstrate more versatility than the other dynamics in reducing the training duration either by reducing the resolution, η (Figure 7A), or increasing the non-linearity, λ (Figure 7B).

**Figure 8:**
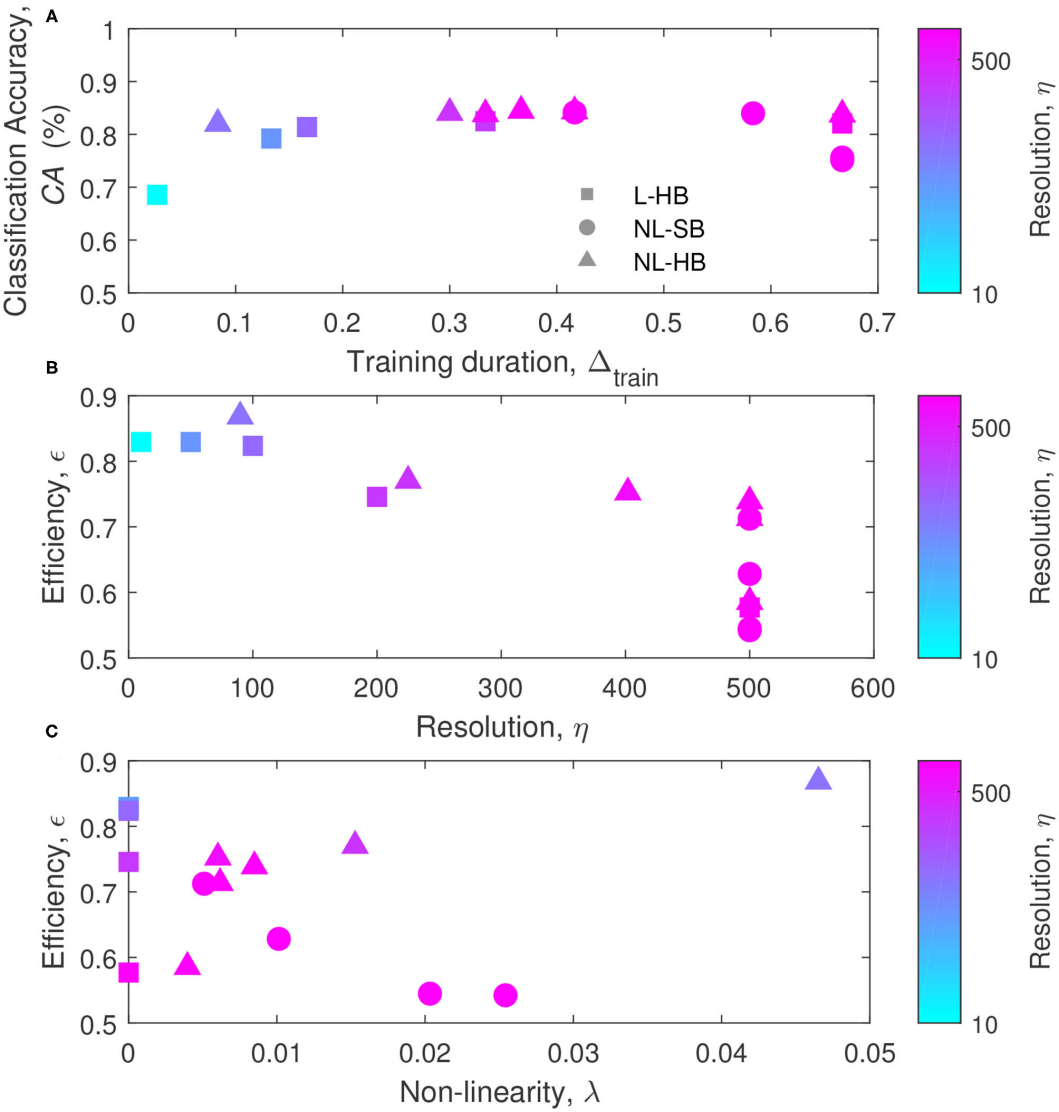
**(A)** Classification accuracy, *CA*, as a function of the training duration, Δ_*train*_, for the various dynamics. **(B)** Efficiency as a function of the resolution, η, and **(C)** efficiency as a function of the non-linearity, λ. The symbols colors follow the resolution value, η, according to the color bars reported on the right sides of the panels.

All the results are summarized in Figure 8. Figure 8A reports the classification accuracy as a function of the training duration, Δ_*train*_, for the various dynamics. The usual increase of *CA* with η is evident for the L-HB case, demonstrating that an increase in synaptic resolution produces a higher classification accuracy at the expense of longer training duration. This fact can be appreciated reminding that the symbol color follows the resolution, η, in agreement with the color bar on the right-hand side of the Figure 8. The saturation visible at high Δ_*train*_ may be just due to the fact that, during training, further *CA* increase takes longer and longer time. In Figure 8A, no general trend can be appreciated for NL-SB and NL-HB synapses. For instance, some NL-SB cases present long training times associated to a degraded *CA* as a consequence of the effect of the non-linearity, according to Figures 4, 7. In addition, for the NL-HB cases, the *CA* shows a limited dependence on Δ_*train*_. In particular, the point corresponding to the lowest training duration, interestingly, guarantees almost the same classification performances as the points requiring a longer training. This case could be considered as the one realizing the best trade-off between classification accuracy and required training time. As a matter of principle, some applications may require both to maximize the *CA* and to minimize Δ_*train*_ (i.e., maximize 1−Δ_*train*_). For this reason, we can define the SNN efficiency, ϵ, as

(7) $$\begin{array}{r} \epsilon =\frac{CA+\left(1-\Delta_{train}\right)}{2}, \end{array}$$

which is normalized between 0 and 1. ϵ values are shown in Figures 8B,C as a function of η and λ, respectively (all the achieved values of the performance metrics and a figure reporting the efficiency as a function of accuracy are reported in the Supplementary Material). The maximum efficiency is reached by the NL-HB case with the lowest resolution and the highest non-linearity (top- and left-most triangle in Figure 8B and top- and right-most triangle in Figure 8C, with η = 90 and λ = 0.047). It corresponds to the dynamics with α = 0.03 in Figure 2C, which grants a classification accuracy that is only slightly affected by resolution and non-linearity, as shown in Figure 4. Such highly non-linear and highly weight-dependent NL-HB dynamics resembles a NL-SB one and may endow the network with longer memory lifetime (Fusi and Abbott, 2007) and a higher synaptic competition within a STDP training framework (van Rossum et al., 2000), resulting in an improved synaptic contrast (right-most filled triangle in Figure 6C). Furthermore, the maximum efficiency dynamics takes advantage of a short training time justified by its low resolution, as shown in Figure 7A. In turn, for the L-HB cases (squares), the efficiency is degraded with increasing resolution as a consequence of the increase of the training duration, as shown in Figure 7A. The non-linearity, instead, deteriorates the efficiency of the NL-SB dynamics (circles in Figure 7C) because it both increases the training duration (Figure 7B) and reduces the classification accuracy (Figure 7).

## 4. Conclusions

In conclusion, we analyzed the impact of the synaptic weight dynamics on the performances of a two-layer fully-connected SNN compatible with a hybrid CMOS/memristive implementation and trained through an unsupervised STDP protocol. We chose weight dynamics that can be realized, at least as a matter of principle, through memristive technology. We found that synapses with non-linear dynamics and hard weight boundary values (NL-HB synapses) give performance advantages for a SNN with STDP-based learning in various aspects. First, NL-HB synapses guarantee the best classification accuracy among the investigated dynamics (see Figures 3, 4, 8A) over all the investigated range of resolution, η. It is worth noticing that this is a significant result in the context of the present literature. Indeed, it has been extensively demonstrated in several publications (Chen et al., 2015; Ambrogio et al., 2018; Fumarola et al., 2018; Moon et al., 2018) that linear synapses enable the best classification accuracy of neuromorphic systems that implement in hardware the back-propagation of the global error. This result has been extended, as a supposedly natural consequence, as holding true for SNNs. However, few recent works from the present authors (La Barbera et al., 2018; Brivio et al., 2019a) have given indications that non-linear synapses can perform better than linear ones for SNNs, which resulted in an interesting debate (Berg et al., 2019). In the present work, we put on firmer and quantitative basis the role of non-linearity on the performances of unsupervised and STDP-based SNNs.

Furthermore, for applications in which the training duration has to be minimized, the NL-HB dynamics also realized the best trade-off between classification accuracy and training duration, in agreement with the mathematical definition of efficiency given above (see Figure 8).

All these results are ascribed to the fact that the NL-HB dynamics produces a distinct behavior of the SNN, with respect to L-HB and NL-SB dynamics. Indeed, in case of hard-bounds, the classification accuracy and the weight contrast (ability to take advantage of a wide portion of the available weight range) is minimally affected by the non-linearity (compare NL-SB and NL-HB cases in Figures 4, 6). Moreover, the non-linearity of NL-HB synapses tends to reduce SNN training duration, in clear opposition with the trend of the soft-bound synapses (Figure 7B). This is the reason for the low training duration for the highly non-linear hard bound synapses, which results in a high efficiency, ϵ, according to the definition above (Figure 8).

In addition, it is interesting to make some considerations from a technological point of view. Memristive devices are characterized by an intrinsic non-linear conductance dynamics. More precisely, we have recently shown that the NL-SB dynamics is the model that faithfully describes the behavior of filamentary memristive devices (Frascaroli et al., 2018; Brivio et al., 2019a). On the other hand, technological efforts have been mainly focused on developing memristive synaptic devices with high resolution and low non-linearity because these are the requirement for hardware neural networks relying on back-propagation of the error. The linear dynamics is usually obtained by truncating the non-linear dynamics in the linear regime. This solution however limits the synapse resolution to a lower values with respect to those that can be obtained with a more complete non-linear dynamics. In fact, in the present study, the dynamics free parameters have been set to realistic values in particular for the non-linear cases. On the contrary, resolutions of 200 and 500 levels can hardly be obtained over a linear conductance evolution (Wang et al., 2016; Bousoulas et al., 2017; Chen et al., 2019). For instance, in one of the best literature results, Wang et al. (2016) reports a nearly linear dynamics over 300 pulses, indicating a resolution close to 300 levels. However, their data is best fitted with a NL-HB models with α = 0.004, γ = 1.02 and a resolution of about 266 levels. Therefore, according to our results, in the case of SNNs with STDP-based unsupervised training, higher classification accuracy values, or efficiency values, can be obtained with non-linear hard-bound synapses relaxing the requirements on resolution and non-linearity for memristive devices. Therefore, high performances for STDP-based SNNs can be obtained with moderately challenging device engineering by embracing, instead of facing, their intrinsic non-linear dynamics. It is worth specifying that simulations have intentionally been performed neglecting any source of variability in the synaptic elements in order to isolate the very effect of synaptic dynamics. From an experimental point of view, the various dynamics may be affected more or less seriously by noise and variability. In particular, we can expect the linear dynamics, being the most challenging in real devices as stated above, to be the most affected by noise and variability. However, a methodological experimental investigation on highly optimized devices is required in order to take into account the different role of dynamics-dependent variability in the simulations.

Finally, the present paper defines a methodology to assess the impact of synaptic dynamics on the performances of a neural network and provides the basis for future works applied to different training protocols, network architectures, applications, and different synaptic dynamics features, e.g., asymmetry between weight depression and potentiation processes and potentially different dynamics evolution, size of the readout layer and, as mentioned above, the impact of dynamics-specific noise and variability features, all of which can have an impact on the trade-offs pointed out in the manuscript.

## Data Availability Statement

The raw data supporting the conclusions of this article will be made available by the authors, without undue reservation.

## Author Contributions

SB conceived the idea and analyzed the data. DL performed the simulations and contributed to data analysis. EV and SS supervised the activity. All authors contributed to the manuscript writing.

## Conflict of Interest

The authors declare that the research was conducted in the absence of any commercial or financial relationships that could be construed as a potential conflict of interest. The reviewer HM declared a past co-authorship with two of the authors SB, SS to the handling editor.
