# Supplementary material for: Non-linear Memristive Synaptic Dynamics for Efficient Unsupervised Learning in Spiking Neural Networks
Source: Front Neurosci. 2021 Feb 1;15:580909. doi: 10.3389/fnins.2021.580909 (PMC7901913; doi:10.3389/fnins.2021.580909)
Supplement: Supplementary file 1 [file Data_Sheet_1.pdf]

frontiers

Supplementary Material

Non-linear Memristive Synaptic Dynamics for Efficient Unsupervised Learning in Spiking Neural Networks

Stefano Brivio [1,*], Denys R. B. Ly [2], Elisa Vianello [2] and Sabina Spiga [1]

[1]CNR - IMM, Unit of Agrate Brianza, Italy;

[2]Univ. Grenoble Alpes, CEA, Leti, F-38000 Grenoble, France

***Correspondence:** Stefano Brivio, stefano.brivio@mdm.imm.cnr.it

1 FORMAL DEFINITION OF THE INVESTIGATED DYNAMICS

We report here and complete the definitions of the investigated dynamics as anticipated in the main manuscript. The linear hard-bound dynamics (L-HB) is defined as

$$\frac{dw_\pm}{dn} = \alpha_\pm, \tag{S1}$$

with $\alpha_\pm \in (0,1]$ and where the $(\cdot)_+$ and the $(\cdot)_-$ stand for potentiation and depression, respectively. Following Fusi and Abbott (2007); Frascaroli et al. (2018); Brivio, S. and Conti, D. and Nair, M. V. and Frascaroli, J. and Covi, E. and Ricciardi, C. and Indiveri, G. and Spiga, S. (2019), the NL-SB equation is given by

$$\begin{cases} \frac{dw_+}{dn} = \alpha_+(1-w)^{\gamma_+} \\ \frac{dw_-}{dn} = -\alpha_- w^{\gamma_-} \end{cases}, \tag{S2}$$

with $\alpha_\pm \in (0,1]$ and $\gamma_\pm \geq 1$. The NL-HB dynamics is the truncated version of the NL-SB properly re-scaled between 0 and 1, as follows

$$\begin{cases} \frac{dw_+}{dn} = \frac{\alpha_+}{w_{stop,+}}(1 - w \cdot w_{stop,+})^{\gamma_+} \\ \frac{dw_-}{dn} = -\frac{\alpha_-}{w_{stop,-}}(w \cdot w_{stop,-} + 1 - w_{stop,-})^{\gamma_-} \end{cases}, \tag{S3}$$

where $\alpha_\pm \in (0,1]$, $\gamma_\pm \in [1,+\infty)$. $N_{stop,\pm}$ are the values of n at which the corresponding NL-SB dynamics is cut to get a NL-HB one. $w_{stop,\pm}$ are the normalization terms that depend on the value of $N_{stop,\pm}$, as

$$\begin{cases} w_{stop,\pm} = 1 - e^{\alpha_\pm N_{stop,\pm}} & for \quad \gamma = 1 \\ w_{stop,\pm} = 1 - \left[1 + \alpha_\pm(\gamma_\pm - 1)N_{stop,\pm}\right]^{1/1-\gamma_\pm} & for \quad \gamma > 1 \end{cases}, \tag{S4}$$

which are different depending on the value of γ because of the difference in the integration of eq. S3.

2 FIGURE OF MERITS OF SYNAPTIC DYNAMICS

The investigated figures of merits are the synaptic resolution, to be also considered as the effective number of levels, η, and the non-linearity, λ. The definition of both is given in the main manuscript. In some cases, the synaptic resolution assumes an analytic form as reported in table S1.

	η
**L-HB**	$\frac{1}{\alpha_\pm}$
**NL-SB**	$\frac{\gamma_\pm+1}{\alpha_\pm}$
**NL-HB** ($\gamma_\pm > 1$)	$\frac{w^2_{stop,\pm}(\gamma_\pm+1)}{\alpha_\pm}\left\{1-\left[1+\alpha_\pm(\gamma_\pm-1)N_{stop,\pm}\right]^{\frac{\gamma_\pm+1}{1-\gamma_\pm}}\right\}^{-1}$
**NL-HB** ($\gamma_\pm = 1$)	$\frac{2w^2_{stop,\pm}}{\alpha_\pm}\left\{1-e^{-2\alpha_\pm N_{stop,\pm}}\right\}^{-1}$

Table S1. Analytical expressions of the resolution, η, for the investigated dynamics.

The definition of the resolution parameter is arbitrary defined to have the possibility to evaluate a finite value also for soft-bound dynamics which reach the boundary values only after an infinite number of pulses. In order to give an intuition of how the estimator of the resolution works, we consider a generic potentiation NL-HB dynamics described by a different equation compared to those used in the main manuscript. The test dynamics is described by the following equation S5:

$$\begin{cases} \frac{dw}{dn} = \alpha e^{-\beta w} & w \in [0,1] \\ \frac{dw}{dn} = 0 & elsewhere \end{cases}, \tag{S5}$$

where α and β are two free parameters. The dynamics are reported in Figure S1 for $\alpha = [1e^{-2}, 3e^{-2}, 1e^{-1}, 3e^{-1}, 1]$ and $\beta = [0.5, 1.5, 2.5]$. Figure S1b,d,f report the same data as Figure S1a,c,e but with a logarithmic x axis. The dynamics have hard bounds that are met when the weight assumes the unitary value. In the figure, the red dots and vertical lines indicate the effective number of levels, η, which are positioned in the same x axis as the dynamics. This graphic method allows for an easy comparison between the η value and the minimum number of pulses that is required to bring the weight from 0 to 1, equivalent to N_{stop}. The figure shows that when the dynamics is roughly linear and, hence, the synaptic levels are about evenly spaced there is no significant difference between η and N_{stop}, (figure S1a-b). Increasing the non-linearity and, thus, accumulating more and more weight steps close to weight boundary, reduces the effective number of levels, thus moving lower and lower the η values away from N_{stop} from Figure S1c-d to Figure S1e-f. These results indicate the given definition of η is at least reasonable.

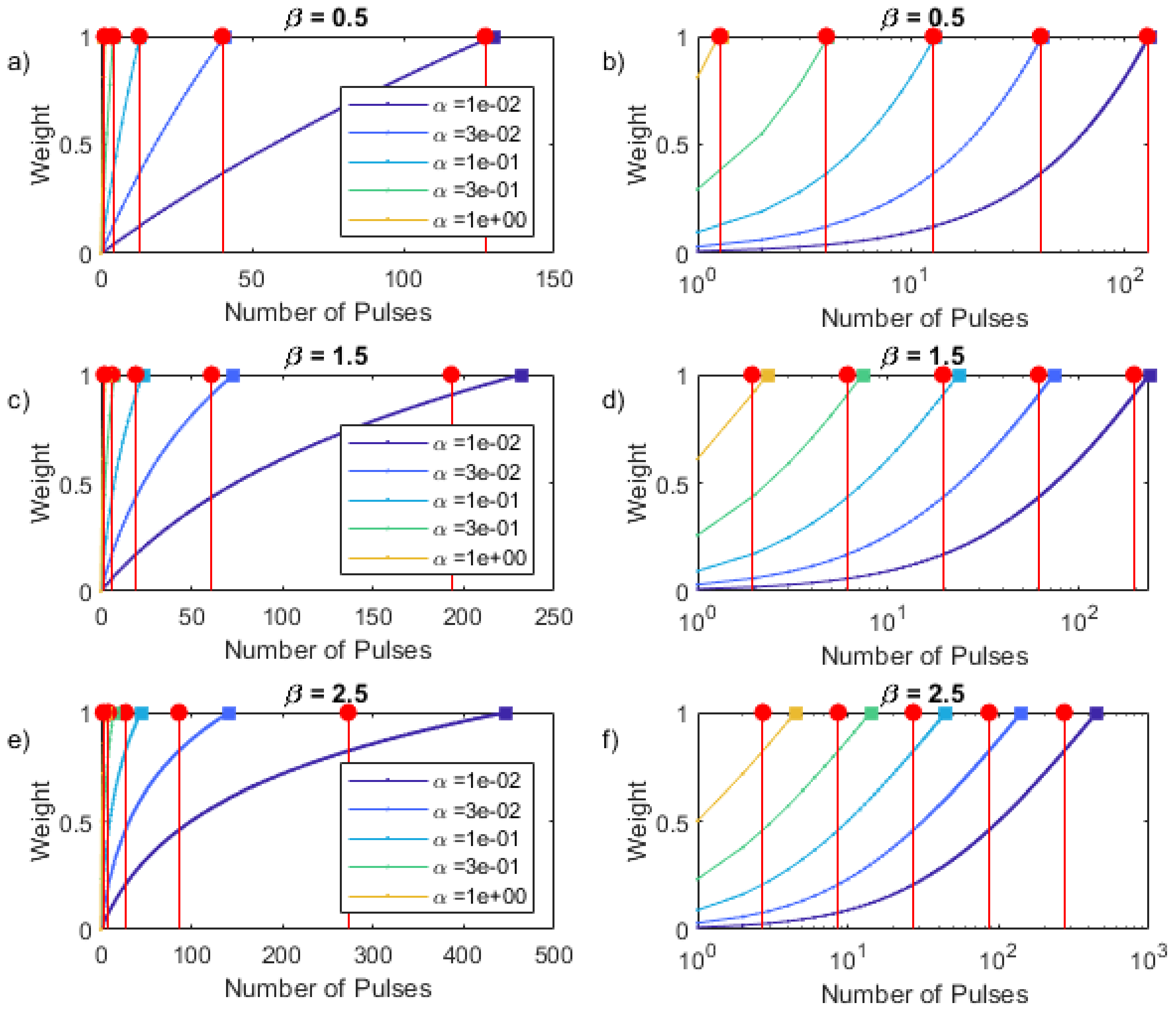

Figure S1. Weight evolution as a function of pulses for the tested dynamics defined by equation S5 for various α and β parameters. Figure S1b,d,f report the same data as Figure S1a,c,e but with a logarithmic x axis.

3 WEIGHT EVOLUTION DURING TRAINING

During SNN training the synaptic weights are tuned according to the learning rule defined in the main manuscript. It is a general fact that, at the end of the training, weight-independent synaptic dynamics result in a bimodal weight distribution, with weight values accumulating at the boundary of the allowed weight range. Conversely, weight-dependent synaptic dynamics tend to accumulate the weight values somewhere in the middle of the allowed weight range. Brivio, S. and Conti, D. and Nair, M. V. and Frascaroli, J. and Covi, E. and Ricciardi, C. and Indiveri, G. and Spiga, S. (2019); Morrison et al. (2007) This fact is well reproduced by our network as reported in Figure S2. For the L-HB cases, final weight distributions show very sharp bimodal distributions, while nonlinear cases show accumulation of weights in intermediate values of the allowed weight range. For the nonlinear case, one distribution peak dominates and small contribution arise as small bumps far away from the main peak (e.g. in Figure S2g-i) or as a shoulder to the main peak (e.g. in Figure S2m-n).

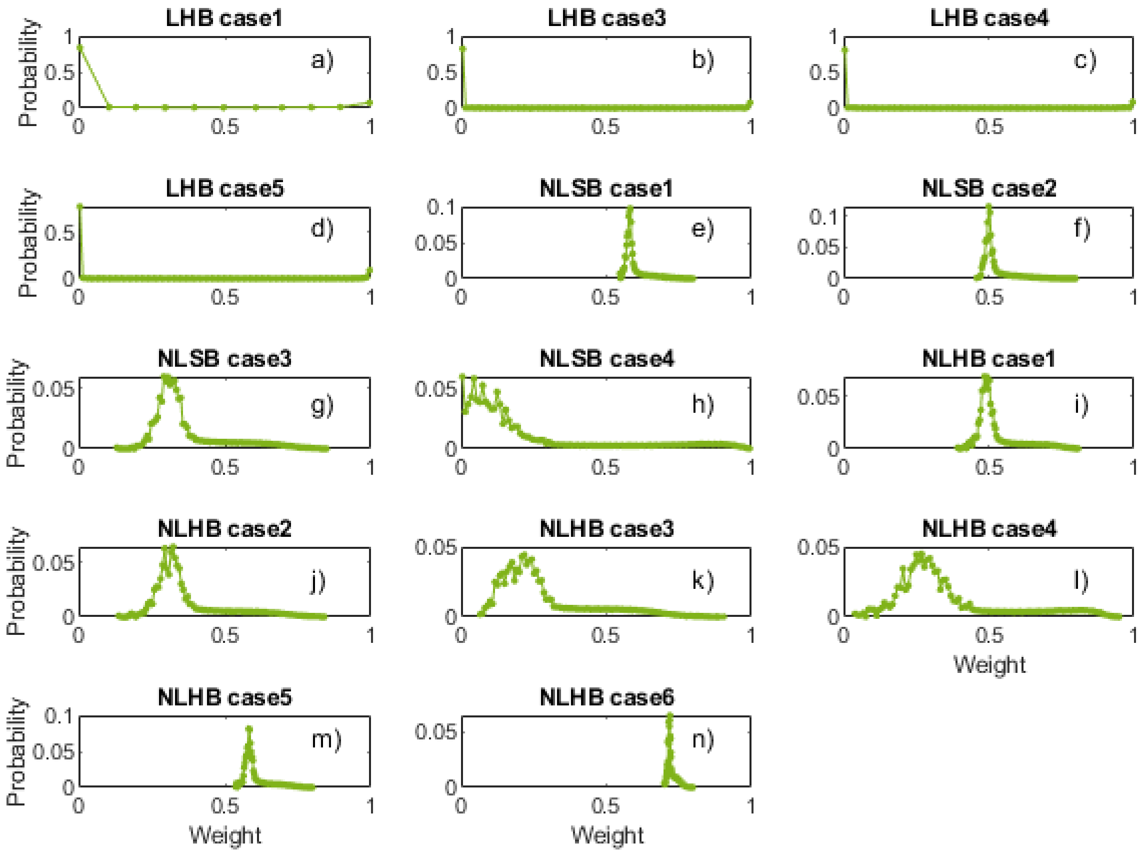

Figure S2. Weight distributions after training for various dynamics cases.

In order to track, during training, the formation of two weight contributions (two peaks), which is fundamental to develop the classification ability, we use different algorithms to identify two clusters. The most appropriate method that we used is the *k-means* algorithms. As a check, the two clusters are identified also by taking the average of the weight values above and below a threshold of 0.5. We name this latter

as ave_{half} algorithm. Furthermore, the two clusters are identified also by taking the average of the weight values above and below a threshold value that is the average of all the synaptic weights. We name this latter as ave_{mean} algorithm. The Figures S3-S5 report the positions of the centers of the two clusters as obtained through the various algorithms for the L-HB, the NL-SB, and the NL-HB synapses, respectively. The results obtained through the *k-means*, the ave_{half}, and the ave_{mean} algorithms are reported as solid line, dashed line and as symbols, respectively. Furthermore, the contrast, i.e. the distance between the centers of the two clusters is reported in black. Figure S3 clearly shows that the training of L-HB synapses gives rise to two well separated distributions and hence, high contrast. Conversely, the nonlinear synapses give rise to low contrast. The contrast is always below 0.5 for the NL-SB case and below 0.4 for the NL-HB case. In any case, it is possible to notice that the three employed algorithms qualitatively agree on the contrast estimation.

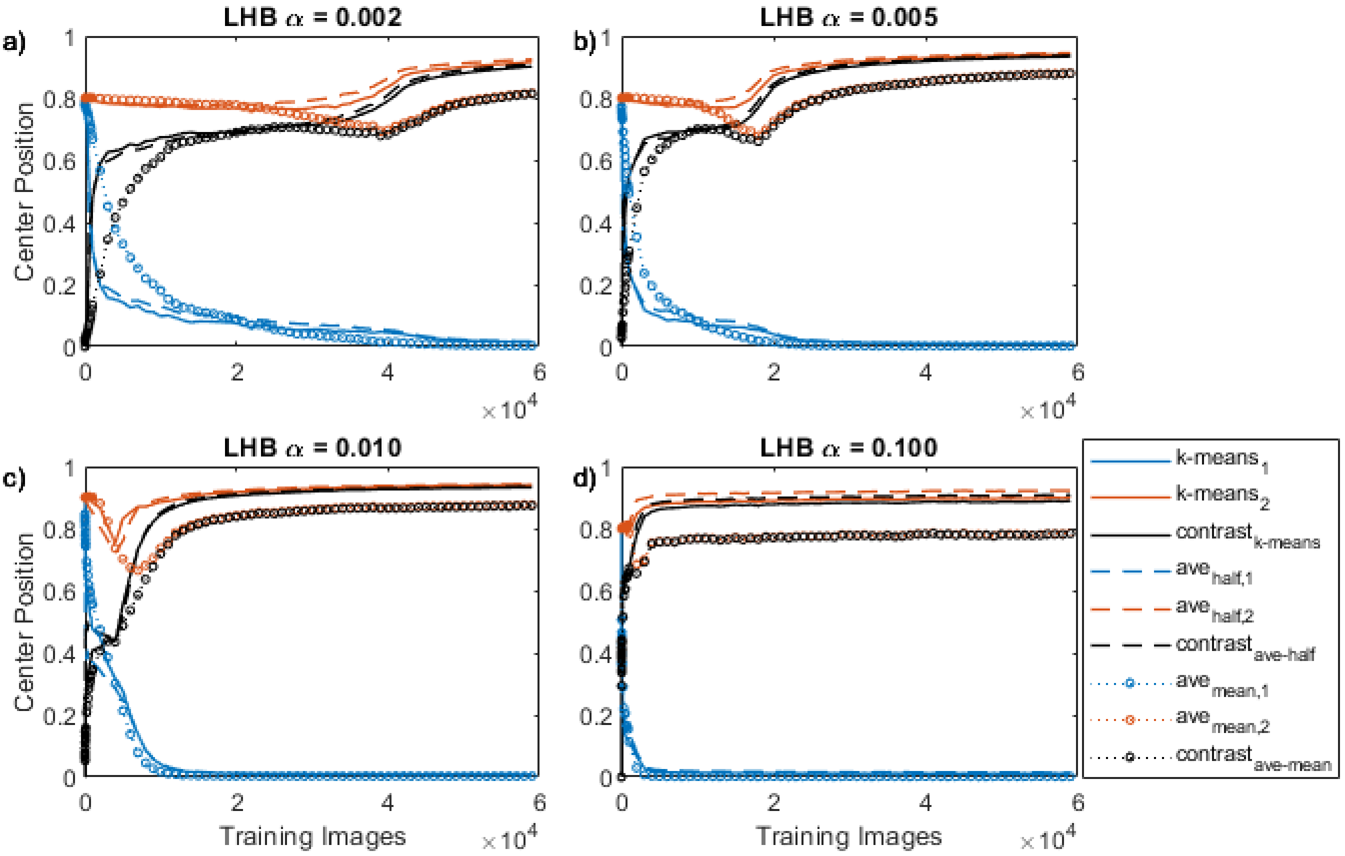

Figure S3. Evolution of the weight of L-HB synapses during training. The figure reports the position of the two weight clusters centers and distance of the two cluster centers (contrast) evaluated with three different algorithms, as described in the text: k-means (solid line), ave_{half} (dashed line), and ave_{mean} (dotted line with symbol). Blue lines correspond to the lower weight cluster centers, orange lines to the higher weight cluster centers, and the black lines to the weight contrast. The quantities are plotted against the number of training images. Panels (a)-(d) report the evolution for different α values.

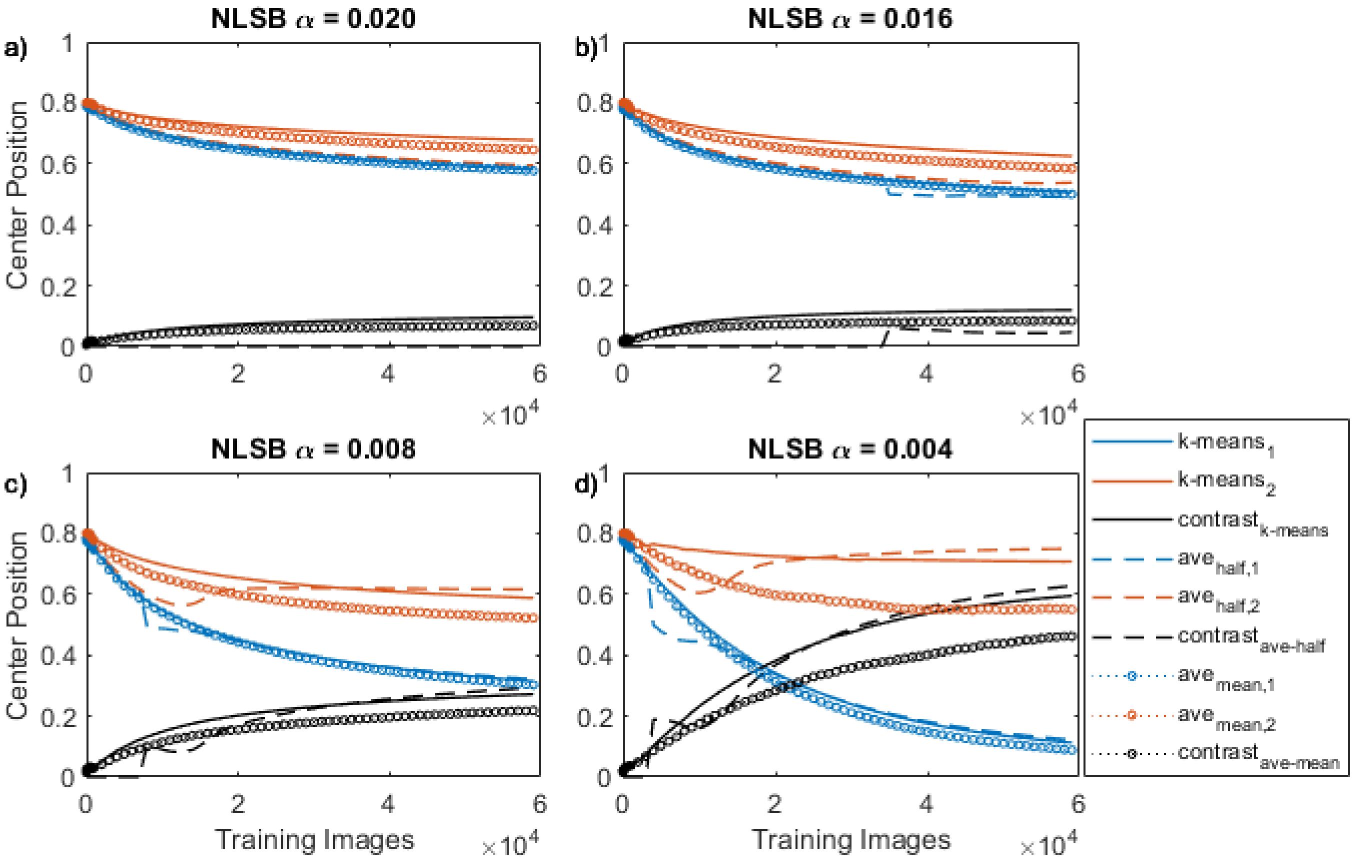

Figure S4. Evolution of the weight of NL-SB synapses during training. The figure reports the position of the two weight clusters centers and distance of the two cluster centers (contrast) evaluated with three different algorithms, as described in the text: k-means (solid line), ave_{half} (dashed line), and ave_{mean} (dotted line with symbol). Blue lines correspond to the lower weight cluster center, orange lines to the higher weight cluster center, and the black lines to the weight contrast. The quantities are plotted against the number of training images. Panels (a)-(d) report the evolution for different α values.

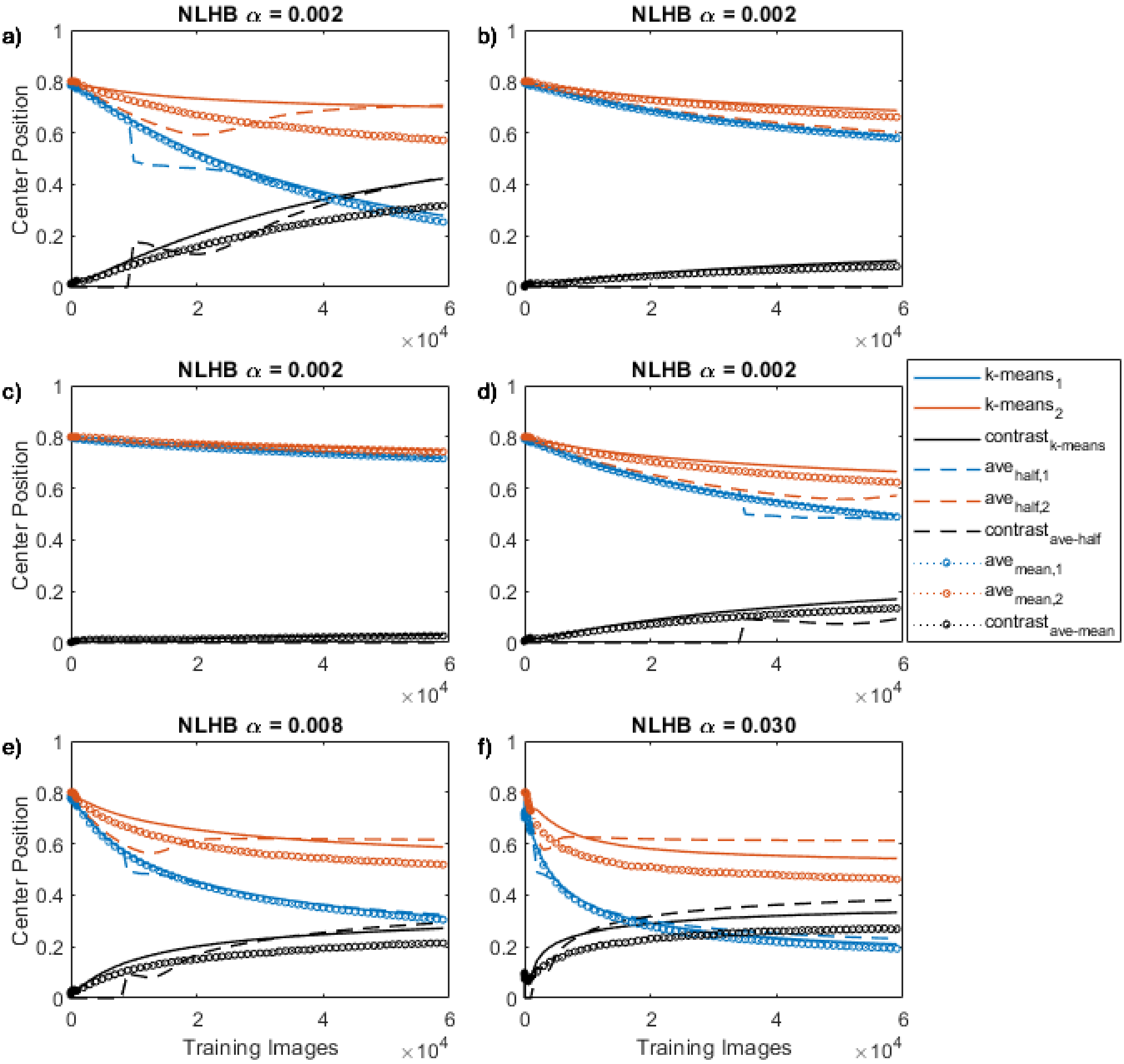

Figure S5. Evolution of the weight of NL-HB synapses during training. The figure reports the position of the two weight clusters centers and distance of the two cluster centers (contrast) evaluated with three different algorithms, as described in the text: k-means (solid line), ave_{half} (dashed line), and ave_{mean} (dotted line with symbol). Blue lines correspond to the lower weight cluster center, orange lines to the higher weight cluster center, and the black lines to the weight contrast. The quantities are plotted against the number of training images. Panels (a)-(d) report the evolution for different α values.

4 NETWORK EFFICIENCY

We report in Figure S6 the network efficiency, as defined in eq. 7 of the manuscript, as a function of the classification accuracy, CA

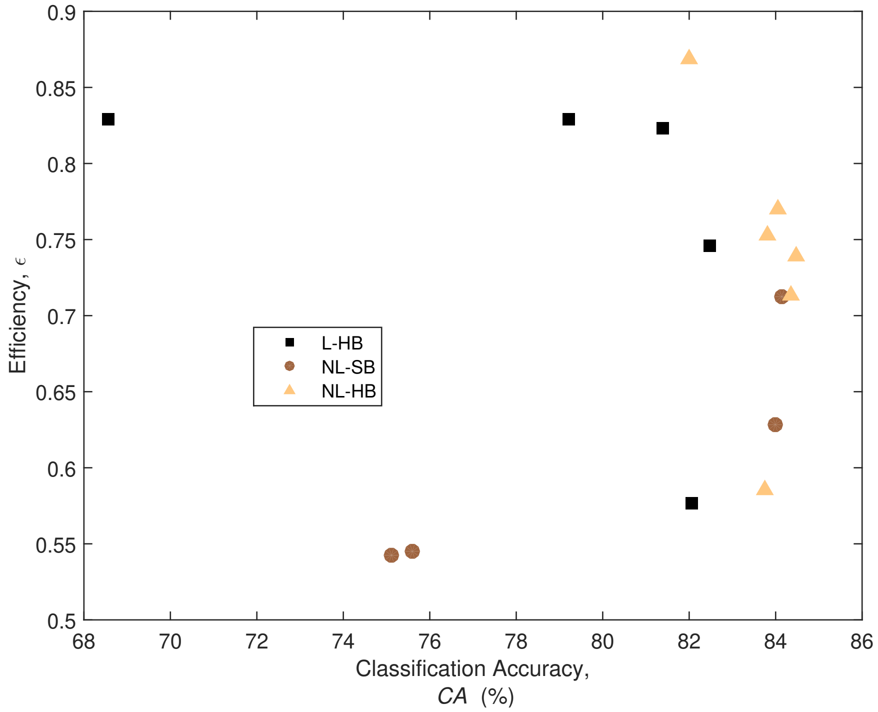

Figure S6. Network efficiency, ϵ, as a function of the classification accuracy, CA, for the various investigated dynamics

5 SUMMARY OF THE RESULTS

The following table S2 collects all the details of the dynamics parameters; corresponding resolution, η, and non-linearity, λ, classification accuracy, CA, training time, Δ_{train}, and efficiency, ϵ, as defined in the main text.

	$\alpha_{\pm}$	$\gamma_{\pm}$	$N_{stop,\pm}$	η	λ	CA (%)	Δ_{train}	ϵ
L-HB	0.1	-	-	10	0	68.6	0.03	0.83
	0.02	-	-	50	0	79.2	0.13	0.83
	0.01	-	-	100	0	81.4	0.17	0.82
	0.005	-	-	200	0	82.5	0.33	0.75
	0.002	-	-	500	0	82.1	0.67	0.58
NL-SB	0.02	9	-	500	0.02	75.1	0.67	0.54
	0.016	7	-	500	0.020	75.6	0.67	0.54
	0.008	3	-	500	0.010	84.0	0.58	0.63
	0.004	1	-	500	0.005	84.2	0.42	0.71
NL-HB	0.002	3	500	402	0.006	83.8	0.33	0.75
	0.008	3	500	225	0.015	84.1	0.30	0.77
	0.03	3	500	90	0.047	82.0	0.08	0.87
	0.002	1.16	559	500	0.004	83.7	0.67	0.59
	0.002	4.57	796	500	0.006	84.4	0.42	0.71
	0.002	9.88	1281	500	0.009	84.5	0.37	0.74

Table S2. List of the investigated dynamics defined by the values of their parameters α, γ, and N_{stop}. The corresponding values for resolution, η, non-linearity, λ, classification accuracy, CA, training time Δ_{train}, and efficiency, ϵ, are also reported.

REFERENCES

Fusi S, Abbott LF. Limits on the memory storage capacity of bounded synapses. *Nature Neuroscience* **10** (2007) 485. doi:10.1038/nn1859.

Frascaroli J, Brivio S, Covi E, Spiga S. Evidence of soft bound behaviour in analogue memristive devices for neuromorphic computing. *Scientific Reports* **8** (2018) 1–12. doi:10.1038/s41598-018-25376-x.

Brivio, S and Conti, D and Nair, M V and Frascaroli, J and Covi, E and Ricciardi, C and Indiveri, G and Spiga, S. Extended memory lifetime in spiking neural networks employing memristive synapses with nonlinear conductance dynamics. *Nanotechnology* **30** (2019) 015102. doi:10.1088/1361-6528/aae81c.

Morrison A, Aertsen A, Diesmann M. Spike-Timing-Dependent Plasticity in Balanced Random Networks. *Neural Computation* **19** (2007) 1437. doi:10.1162/neco.2007.19.6.1437.
